# Genetic variation and response to selection of photosynthetic and forage characteristics in Kentucky bluegrass (*Poa pratensis* L.) ecotypes under drought conditions

**DOI:** 10.3389/fpls.2023.1239860

**Published:** 2023-11-10

**Authors:** Nikwan Shariatipour, Zahra Shams, Bahram Heidari, Christopher Richards

**Affiliations:** ^1^ Department of Plant Production and Genetics, School of Agriculture, Shiraz University, Shiraz, Iran; ^2^ Department of Horticulture Science, School of Agriculture, Shiraz University, Shiraz, Iran; ^3^ United States Department of Agriculture, The Agricultural Research Service, National Laboratory for Genetic Resources Preservation, Fort Collins, CO, United States

**Keywords:** ecotype, forage yield, genetic advance, Kentucky bluegrass, photosynthesis

## Abstract

**Introduction:**

Evaluation of the effects of water-limited conditions on the photosynthetic characteristics and forage yield is important for enhancing the forage productivity and drought tolerance in Kentucky bluegrass (*Poa pratensis* L.).

**Methods:**

In the present study, 100 P*. pratensis* ecotypes collected from different geographical areas in Iran were assessed under well-watered and drought stress conditions. Genetic variation and response to selection for the photosynthetic characteristics [i.e., net photosynthesis rate (A), stomatal conductance (*g_s_*), transpiration rate (T_r_), chlorophyll content (Chl), and photochemical efficiency (Fv/Fm)] and forage yield [fresh forage yield (FY) and dry forage yield (Dy)] traits were analyzed during the 2018 and 2019 growing seasons.

**Results and discussion:**

Drought stress had negative effects on evaluated photosynthesis parameters and significantly reduced dry and fresh forage yields. On average, FY with a 45% decrease and *g_s_* with a 326% decrease under drought stress conditions showed the highest reduction rate among forage yield and photosynthesis traits, respectively. Genotypic coefficients of variation (GCV) for FY were lower under drought stress. The estimates of heritability, genetic advance, and genetic advance as percentage of mean showed the predominance of additive gene action for the traits. Overall, the results showed that “Ciakhor”, “Damavand”, “Karvandan”, “Basmenj”, “Abr2”, “Abrumand”, “Borhan”, “Hezarkanian”, “LasemCheshmeh”, “Torshab”, and “DoSar” have higher forage yield production with little change between two irrigation regimes, which makes them promising candidates for developing high-yielding drought-tolerant varieties through breeding programs.

## Introduction

Nowadays, climate changes that severely affect plant growth shift from monsoon patterns and global warming to drought more intensely and frequently. As a detrimental abiotic stress for plant growth, drought threatens production in agriculture in most countries and geographical regions (Liu et al., 2022a). Drought has periodically affected agricultural productivity in Iran, which is one of the countries suffering from low precipitation and water shortages. Iran’s climate, with the exception of the northern coastal areas and western parts, is mainly arid and semi-arid, with high temperatures up to +50°C and 240 mm average annual rainfall (Heshmati, 2007; Amiri and Eslamian, 2010). Such conditions can lead to shortage of water resources and additional challenges for water distribution that can limit crop production in Iran (Noorisameleh et al., 2020).

Crop production losses caused by drought are the most important and damaging of all abiotic stresses (Seleiman et al., 2021). Photosynthesis plays a central role in plant growth and crop productivity and has become a major focus of research on abiotic stress (Gururani et al., 2015; Kebbas et al., 2015; Li et al., 2015; Liu et al., 2017; Fang et al., 2018; Zhang et al., 2022). The stomatal (stomatal closure due to decreased CO_2_ intake) or nonstomatal (low photosynthetic rate in mesophyll tissue) responses, or both, are considered as the main factors responsible for decreased photosynthesis during drought stress (Varone et al., 2012; Ghotbi-Ravandi et al., 2014). Stomatal closure that restricts the diffusion of CO_2_ into the mesophyll of leaves is an essential response to decrease evaporative water loss (Cornic, 2000). Evaluation of adaptive photosynthetic responses of plants can facilitate breeding efforts directed toward developing tolerant varieties for challenging and water-limited environmental conditions (Fahad et al., 2015; Saud et al., 2014; Torres-Ruiz et al., 2015; Liang et al., 2020; Yang et al., 2021).

Kentucky bluegrass (*Poa pratensis* L.) as a perennial grass with good spring green-up and forage quality is well suited for animal grazing. The grazing tolerance of this plant species is better than other cool-season forage grasses, which makes it an ideal species for permanent pastures. Kentucky bluegrass, unlike most cool season grasses, spreads by rhizomes, which helps it fill in open areas and produce a denser sod, which makes it ideal for erosion control. In addition, *P. pratensis* is more drought tolerant than many other grass species, which makes it a suitable candidate forage crop in arid and semi-arid areas. Previous studies determined that *P. pratensis* has a native distribution that spans different climatic regions of Iran, especially in the western and northern regions along the Zagros and Alborz Mountain ranges (Shariatipour et al., 2022; Ghanbari et al., 2023). The profusion of potential Kentucky bluegrass ecotypes provides high phenotypic and genotypic diversity for better stability against the adverse climate change effects (Shariatipour et al., 2022). Better understanding of differential physiological responses to water-limited conditions is important for the unraveling stress tolerance mechanisms and managing breeding strategies to identify stress-tolerant Kentucky bluegrass genotypes. In the Zhang et al. (2019) study, the “Wildhorse” cultivar of Kentucky bluegrass was exposed to drought stress and results indicated that drought stress led to cell membrane damage, resulting in decline in photosynthetic rate, chlorophyll content, and visual quality in Kentucky bluegrass. In another study, the contribution of silicate in the photosynthesis regulation and related metabolic pathways was investigated in Kentucky bluegrass (cv. “Arcadia”) tested under drought stress (Saud et al., 2016). Additionally, the effect of foliar application of cytokinin and potassium on stimulation of stomatal opening and resumption of photosynthesis in the recovery process of Kentucky bluegrass plants exposed to long-term drought stress was investigated (Hu et al., 2013). Analysis of genetic variation in Kentucky bluegrass has shown that simultaneous selection may be possible for important characters for the development of superior turf types (Berry et al., 1969). Results of the Wang and Huang (2003) study demonstrated the genotypic variation for abscisic acid (ABA) accumulation and physiological parameters in four cultivars of Kentucky bluegrass tested under drought stress. In the Merewitz et al. (2010) study, evaluation of agronomic traits and recovery of Kentucky bluegrass genotypes demonstrated variation in response of genotypes to drought stress and the potential for the development of hybrids with improved drought tolerance and performance during recovery. However, most previous studies focused on the agronomy of this species as a turf grass and did not assess the role of genetic variation of photosynthesis activities in response to drought stress in Kentucky bluegrass. Therefore, our objectives were to (1) analyze the effects of drought stress on photosynthesis parameters and forage yield traits; and (2) estimate genetic variation, heritability, and efficiency of response to selection of photosynthetic variation for the improvement of drought tolerance in a collection of Kentucky bluegrass ecotypes from Iran.

## Materials and methods

### Plant material

Plan material consisted of 176 wild Kentucky bluegrass ecotypes that were collected from different geographical areas in Iran (Shariatipour et al., 2022). The collected samples are not threatened species in Iran and were identified following the NCBI Taxonomy descriptions (https://www.ncbi.nlm.nih.gov/Taxonomy/Browser/wwwtax.cgi?lvl=0&id=4545). Each clone sample containing 10 to 15 tillers was collected from a depth of 40 cm of soil and transferred to plastic pots for clonal propagation in a greenhouse. After pre-evaluation of the whole population, 100 viable accessions were selected for growing in the field and further phenotypic evaluation.

### Experimental design and drought stress treatment

**Figure 1:**
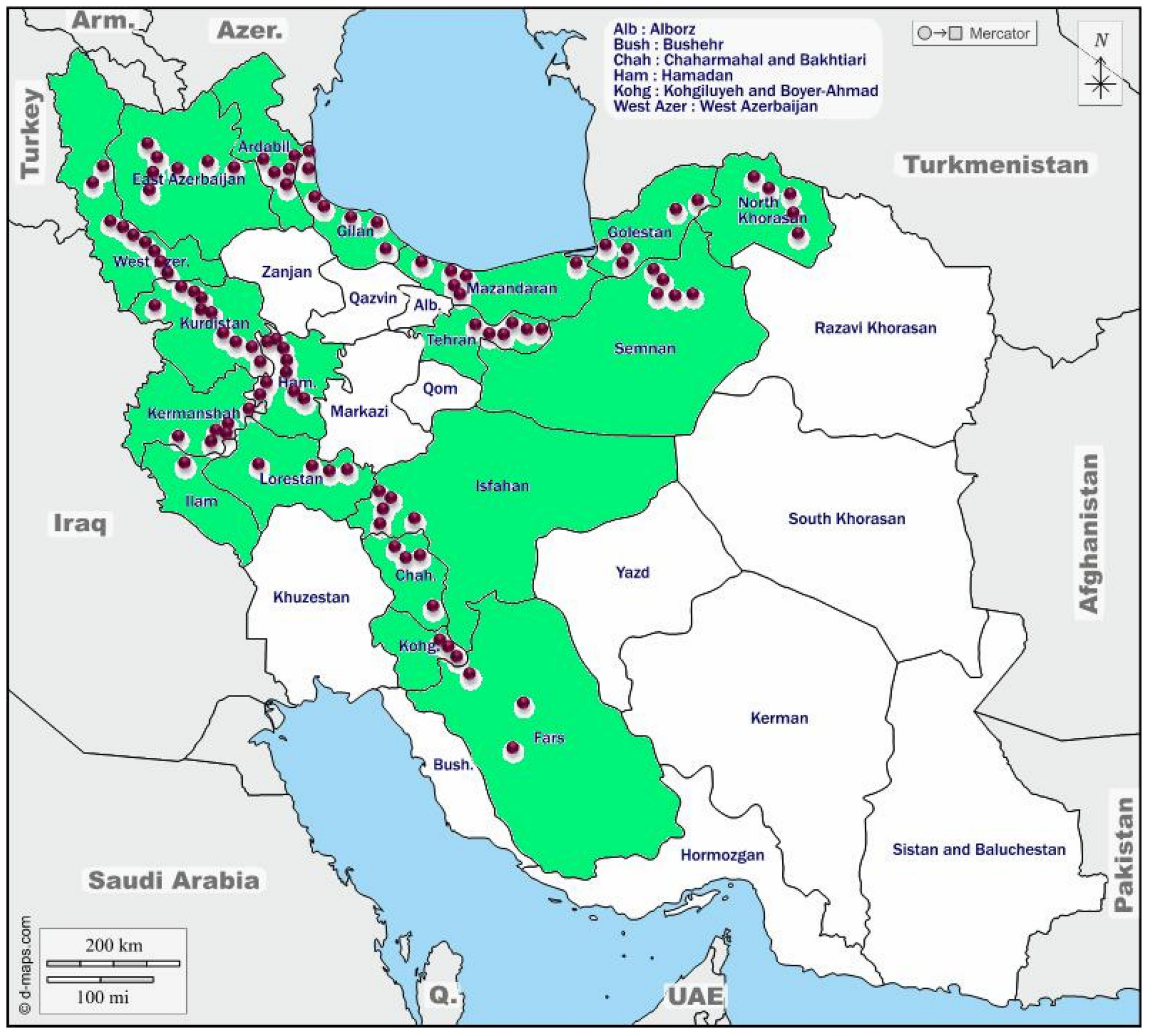
The collection areas for *Poa pratensis* accessions in Iran. The green color indicates provinces and the purple circles represent the approximate location of the collected accessions.

The rhizomes of the selected accessions were grown in the Shiraz University field research station at Bajgah (52° 35 N and 39° 4 E, 1,810 m) over 2 years (2017–2018 and 2018–2019 seasons). The geographical information about the areas where the accessions collected is presented in **Figure 1** and **Supplementary Table S1**. The long-term mean of maximum (22.95°C) and minimum (4.9°C) temperatures and mean annual precipitation of 394 mm generally without rain during the summer made supplemental irrigation necessary for growing the crop.

After field establishment, the germplasm panel was subjected to two irrigation regimes, one as a control with irrigation over the crop growing cycle and one as a drought stress treatment. The experiment was established in a randomized complete block design (RCBD) with two replications in each irrigation treatment. Each plot in the RCBD design contained one clone with a distance of 80 cm between clones. The plants continued to grow in the second year. The soil information used in the current study is shown in **Table 1**. The data showed that the soil had a clay loam texture. The soil water content ( θ i ) in the root zone was measured to determine the net irrigation depth ( $d_{n}$ ) following Eq. 1 (Israelsen and Hansen, 1962):

**Table 1:** The physical characteristic of soil in the field used for evaluation of genetic diversity in *Poa pratensis* accessions.

Parameter	Unit	Soil depth (cm)	
		0-30	30-60
Field capacity (FC) (−0.033 MPa)	cm^3^ cm^−3^	32	33
Permanent wilting point (PWP) (−1.5 MPa)	cm^3^ cm^−3^	11	16
Bulk density (BD)	g cm^−3^	1.31	1.37
Clay	%	36	39
Sand	%	25	27
Silt	%	39	34
Texture	–	Clay loam	Clay loam


(1) $$d_{n}=\sum_{i=1}^{n}\left(\theta_{FCi}-\theta_{i}\right)\times \text{Δ}z_{i}$$

where

$\theta_{FCi}$ = the volumetric soil water content in layer *i* at field capacity

*n* = the number of soil layers

$d_{n}$ = the net irrigation water depth (m)

$\theta_{i}$ = the volumetric soil water content in layer *i*

$\text{Δ}z_{i}$ = the thickness of soil in layer *i* (m)

Based on soil characteristic, *n* and *i* are considered 1 in this equation. Field capacity data were used for the irrigation efficiency of 90%, equal to 10% water loss, which was used to gain the gross irrigation water ( $d_{g}$ ) based on Eq. 2 (Israelsen and Hansen, 1962):


(2) $$d_{g}=d_{n}/0.9$$

then, the 50% and 100% $d_{g}$ were applied for drought stress and non-stress treatments, respectively (Israelsen and Hansen, 1962). Soil water content was constant in 2 years and a drip irrigation system with a weekly irrigation frequency was followed.

### Measurements

#### Photosynthetic rate (A), stomatal conductance (*g*_s_), and transpiration rate (T_r_)

Four weeks after the rhizome establishment in the field and implementing drought stress treatment, all photosynthesis-related traits were measured in both irrigation regimes. Single-leaf A, *g*_s_, and T_r_ were measured at 12:00–14:00 during sunny days in 10 to 12 whole fully expanded leaves using the LCi portable full-automatic photosynthetic measurement system (ADC Bio-Scientific, Ltd., Hertfordshire, UK). After stabilization in the chamber, all photosynthetic parameters of the leaves in each sample were recorded in 2-min intervals by the device All records were performed at 800 μmol m^−2^ s^−1^ photosynthetic photon flux density, which was the light saturation point for Kentucky bluegrass leaves as described by Saud et al. (2014).

#### Photochemical efficiency (Fv/Fm)

Expanded leaves were used for leaf photochemical efficiency as the ratio between variable and maximum fluorescence (Fv/Fm) in the non-energized state accomplished by exposure to darkness. After adaptation of selected leaves to darkness for 30 min, measurements were made on intact leaves with a chlorophyll fluorescence meter (Chlorophyll Fluorometer, OS-30p, Opti-sciences, Inc., USA). The light intensity for the readings was 3,500 μmol.

#### Chlorophyll content

Four weeks after implementing drought stress treatment, chlorophyll content was measured by soaking the expanded leaves (0.1 g) in dimethyl sulfoxide solution at 40°C for 48 h in plants tested under both irrigation treatments. Absorbance of the extracts was read out at 663 and 645 nm wavelength using a spectrophotometer (Epoch Microplate Spectrophotometer, BioTek Instruments, Inc., USA). These are expressed as mg g^−1^ dry leaf weight (Fu and Huang, 2001).

#### Forage yield traits

Forage yield was expressed as fresh- and dry-weight yield. FY was measured as the weight of fresh herbage harvested per plot, and after drying at 72°C for 48 h, the measured weight was expressed as DY.

#### Statistical and biometrical–genetic analyses

Analysis of variance (ANOVA) for the RCBD was carried out to examine significance of the years, irrigation regime (non- and drought stress), genotype effects and their interactions. The residual and predicted values for each trait were subjected to the ANOVA assumptions test. The expected mean squares of the general linear model (GLM) were used for variance component estimation (**Table 2**). In GLM, the effect of year was random, whereas accession and irrigation regime were fixed.

**Table 2:** Expected mean squares for photosynthetic parameters and forage yield traits across two environments (non- and drought stress) in *Poa pratensis* accessions.

Source of variation	Degree of freedom	Expected mean squares
Block	*r* − 1 = 1	
Genotype	*g* − 1 = 99	$\sigma_{e}^{2}+r\sigma_{g}^{2}$
Error	(*r* − 1) (*g* − 1) = 99	$\sigma_{e}^{2}$

g, genotype; r, number of blocks; $\sigma_{e}^{2}$ , error variance; $\sigma_{g}^{2}$ , genotypic variance.

The phenotypic, genotypic, and environmental variances were estimated according to the expected value of mean square of the sources of variations in the ANOVA table described by Federer and Searle (1976) as follows (equation (Eqs. 3–5):


(3) $$\sigma_{\text{g}}^{2}=\frac{{\text{MS}}_{\text{g}}-{\text{MS}}_{\text{e}}}{\text{r}}$$

(4) $$\sigma_{\text{e}}^{2}={\text{MS}}_{\text{e}}$$

(5) $$\sigma_{\text{P}}^{2}=\sigma_{\text{g}}^{2}+\sigma_{\text{e}}^{2}$$

where MS_g_, MS_e_, and r are genotypic mean square, error mean square, and the number of replications, respectively.

Phenotypic (PCV) and genotypic (GCV) coefficients of variation were estimated according to Burtone and De Vane (1953) (Eqs. 6 and 7):


(6) $$\text{PCV}=\frac{\sqrt{\sigma_{p}^{2}}}{\mu }\times 100$$

(7) $$\text{GCV}=\frac{\sqrt{\sigma_{g}^{2}}}{\mu }\times 100$$

where μ is the mean of population for the tested traits. The broad-sense heritability (h^2^) which shows the contribution of the genetic variance in the phenotypic variation of a trait, was calculated according to method of Lush (1940) (Eq. 8):


(8) $${\text{h}}^{2}=\frac{\sigma_{\text{g}}^{2}}{\sigma_{\text{p}}^{2}}$$

In the above equations, $\sigma_{P}^{2}$ , $\sigma_{\text{g}}^{2}$ and $\sigma_{\text{e}}^{2}$ stand for the phenotypic, genotypic, and environmental variances, respectively.

The genetic advance (GA) and genetic advance as percentage of trait mean (GAM) were estimated according to Johnson et al. (1955) (Eqs. 9 and 10):


(9) $$\text{GA}=\text{k}\times \sigma_{\text{e}}\times \frac{{\text{h}}^{2}}{100}$$

(10) $$\text{GAM}=\frac{\text{GA}}{\text{μ}}\times 100$$

where the constant k is the standardized selection differential or selection intensity. The value of k at 5% proportion selected is 2.063.

The phenotypic and genotypic correlation coefficients were calculated (Eqs. 11 and 12) to determine the relationship of traits.


(11) $${\text{r}}_{\text{p}\left(\text{XY}\right)}=\frac{{\text{S}}_{\text{p}\left(\text{XY}\right)}}{{\text{S}}_{\text{p}\left(\text{X}\right)}\times {\text{S}}_{\text{p}\left(\text{Y}\right)}}$$

(12) $${\text{r}}_{\text{g}\left(\text{XY}\right)}=\frac{{\text{S}}_{\text{g}\left(\text{XY}\right)}}{{\text{S}}_{\text{g}\left(\text{X}\right)}\times {\text{S}}_{\text{g}\left(\text{Y}\right)}}$$

where ${\text{r}}_{\text{p}\left(\text{XY}\right)}$ , ${\text{S}}_{\text{p}\left(\text{XY}\right)}$ , ${\text{S}}_{\text{p}\left(\text{X}\right)}$ , ${\text{S}}_{\text{p}\left(\text{Y}\right)}$ , ${\text{S}}_{\text{g}\left(\text{XY}\right)}$ , ${\text{r}}_{\text{g}\left(\text{XY}\right)}$ , ${\text{S}}_{\text{g}\left(\text{Y}\right)}$ , and ${\text{S}}_{\text{g}\left(\text{X}\right)}$ are the phenotypic correlation between traits X and Y, the phenotypic covariance between traits X and Y, the root of the phenotypic variance of trait X, the root of the phenotypic variance of trait Y, the genotypic correlation between traits X and Y, the genotypic covariance between traits X and Y, the root of the genotypic variance of trait Y, and the root of the genotypic variance of trait X, respectively. The key photosynthetic parameters associated with forage yield traits were determined using stepwise regression (Montgomery, 2006). A heatmap clustering was constructed based on the ward.D2 linkage algorithm and Manhattan distance metrics. Statistical analyses were performed using SAS version 9.4 (SAS Institute, Cary, NC, USA) and R *TraitStats* (Nitesh et al., 2020), *corrplot* (Wei et al., 2017), and *pheatmap* (Kolde and Kolde, 2018) packages.

## Results

### Analysis of variance and change of photosynthesis and forage yield traits under the well-watered and drought stress conditions

**Figure 2:**
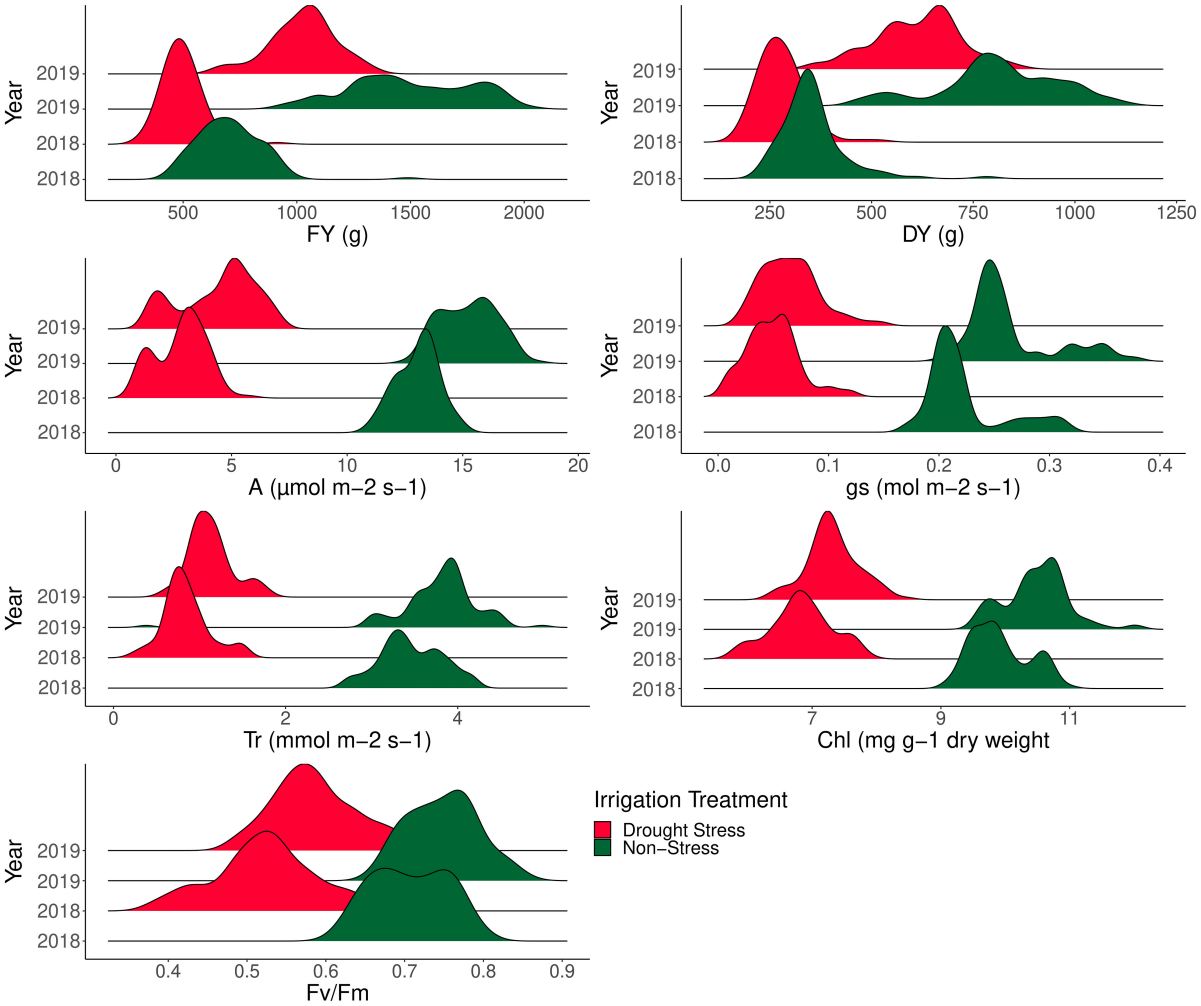
Phenotypic variation of 100 *Poa pratensis* accessions evaluated in non-stress and drought stress conditions over 2018 and 2019.

ANOVA was performed to assess the effects of year, genotype, irrigation regime, and their interactions following mean comparison for photosynthesis and forage-related traits (**Table 3**). Prior to ANOVA, the test of ANOVA assumptions indicated the additive effects of the components in the model. The results of ANOVA showed that main and interaction effects were significant for the traits (**Table 3**). Results of mean comparisons of photosynthetic parameters and forage yield traits for the two environments over the years are presented in **Figure 2**. Drought stress reduced forage yield and photosynthetic traits in both years. In addition, Kentucky bluegrass accessions had higher quantity for assessed traits especially for FY and DY in the second year (**Figure 2**). The FY with 43% and 46% losses was considerably reduced under drought stress in 2018 and 2019, respectively. The DY trait showed 24% and 29% (2019) reductions under drought -stress treatment in 2018 and 2019, respectively (**Figure 2**, **Table 4**). Photosynthetic traits especially A, *g_s_*, and T_r_ showed high reduction in response to drought stress (**Figure 2**, **Table 4**). Analysis of distribution of traits showed that the genotypes had higher phenotypic variation for FY and DY in the second than in the first year. However, the genotypes represented relatively similar phenotypic variation for photosynthesis phenotypes over the 2 years (**Figure 2**). Evaluation of traits over treatments showed inconsistent response to irrigation treatments. Large differences in response of genotypes to irrigation treatments was observed for *g_s_* where the genotypes had higher variation for *g_s_* in drought stress treatment than in normal irrigation conditions (**Figure 2**). The highest decrease in photosynthesis-related traits belonged to A, which was 363.35% under drought stress in the first year, followed by *g_s_* (346.56%) and T_r_ (309.98%). The Fv/Fm showed a lower increase (26.60%) while *g_s_* with a 305.72% decrease showed higher reduction among photosynthetic traits under drought stress in the second year, followed by A (245.01%) and T_r_ (235.88) (**Figure 2**, **Table 4**).

**Table 3:** Source of variations and mean squares in combined analysis of variance (cANOVA) for traits assessed in non-stress and drought stress in 100 *Poa pratensis* accessions in 2018 and 2019.

Characters	Irrigation regime (Ir, df = 1)	Error I (df = 2)	Genotype (G) (df = 99)	(Ir × G) (df = 99)	Error II (df = 198)	Year (Y) (df = 1)	Y × Ir (df = 1)	Y × G (df = 99)	Y × G × Ir (df = 99)	Residual (df = 200)
FY	23,271,035.52^***^	5,115.25	116,521.07^***^	45,513.89^***^	5,482.94	87,757,778.53^***^	3,314,064.13^***^	39,434.88^***^	40,753.07^***^	3,020.60
DY	3,162,499.07^***^	898.29	50,298.39^***^	14,875.64^***^	2,200.59	31,668,496.63^***^	657,526.96^***^	15,778.87^***^	11,865.47^***^	1,711.00
A	22,172.99^***^	3.46	7.06^***^	1.79^***^	0.19	725.98^***^	18.04^***^	1.76^***^	1.47^***^	0.16
*g_s_*	6.923^***^	0.011	0.005^***^	0.0021^***^	5.3 E-5	0.168^***^	0.036^***^	4.4 E-4^***^	4.5 E-4^***^	4.8 E-5
T_r_	1,392.70^***^	0.384	0.644^***^	0.213^***^	0.008	18.650^***^	0.094^***^	0.092^***^	0.098^***^	0.005
Chl	1,949.94^***^	3.22	0.98^***^	0.33^***^	0.04	54.76^**^	0.35^***^	0.18^***^	0.19^***^	0.032
Fv/Fm	5.82^***^	0.05	0.015^***^	0.006^***^	4.5 E-4	0.61^***^	0.036^***^	0.0011^***^	0.0010^***^	2.9 E-4

** and *** represent non-significant and significant at p< 0.01 and p< 0.001, respectively. Ir, irrigation regime; G, genotype; Y, year, Error I = R(M); Error II = R × G(M); FY, forage fresh yield; DY, forage dry yield; A, net photosynthetic rate; g_s_, stomatal conductance; T_r_, transpiration rate; Chl, chlorophyll content; Fv/Fm, photochemical efficiency.

**Table 4:** Mean value, phenotypic coefficients of variation (PCV), genotypic coefficients of variation (GCV), broad-sense heritability ( ${\text{h}}_{b}^{2}$ ), genetic advance (GA) and genetic advance as percentage of mean (GAM) of studied traits measured from 100 accessions of *Poa pratensis* evaluated in non-stress and drought stress environments during years 2018 and 2019.

Trait	Mean ± SE (2018)		Non-stress*									
			GCV (%)		PCV (%)		${\text{h}}_{b}^{2}$ (%) ± SE		GA		GAM (%)	
	Non-stress condition	Drought stress	2018	2019	2018	2019	2018	2019	2018	2019	2018	2019
FY	704.91^a^ ± 11.08	492.53^b^ ± 6.33	19.62	17.54	22.26	18.23	77.70	92.58	251.19	520.23	35.63	34.77
DY	349.11^a^ ± 6.06	280.70^b^ ± 4.76	19.97	19.35	24.61	20.19	65.83	91.88	116.51	307.41	33.37	38.22
A	13.04^a^ ± 0.06	2.82^b^ ± 0.08	6.43	7.72	7.02	8.23	83.87	88.09	1.58	2.28	12.13	14.93
*g_s_*	0.22^a^ ± 0.002	0.05^b^ ± 0.002	15.84	15.23	16.23	15.53	95.21	96.28	0.07	0.08	31.83	30.79
T_r_	3.46^a^ ± 0.02	0.84^b^ ± 0.02	10.26	13.33	10.51	13.47	95.37	97.94	0.71	1.03	20.64	27.18
Chl	9.92^a^ ± 0.03	6.84^b^ ± 0.04	4.37	4.55	4.73	4.82	85.36	89.24	0.83	0.93	8.32	8.86
Fv/Fm	0.71^a^ ± 0.003	0.52^b^ ± 0.005	6.48	5.50	6.94	6.05	87.06	82.72	0.09	0.08	12.45	10.31
Trait	Mean ± SE (2019)		Drought stress*									
			GCV (%)		PCV (%)		${\text{h}}_{b}^{2}$ (%) ± SE		GA		GAM (%)	
	Non-stress condition	Drought stress	2018	2019	2018	2019	2018	2019	2018	2019	2018	2019
FY	1,496.05^a^ ± 19.24	1,026.22^b^ ± 10.59	14.25	13.67	18.21	14.62	61.22	87.33	113.10	269.98	22.96	26.31
DY	804.37^a^ ± 11.46	621.29^b^ ± 7.73	19.48	16.43	24.01	17.64	65.82	86.80	91.36	195.94	32.55	31.54
A	15.25^a^ ± 0.09	4.42^b^ ± 0.12	36.11	36.29	39.81	37.31	82.27	94.59	1.90	3.21	67.46	72.70
*g_s_*	0.26^a^ ± 0.003	0.07^b^ ± 0.002	46.54	39.66	47.18	41.52	97.34	91.25	0.05	0.05	94.60	78.05
T_r_	3.79^a^ ± 0.02	1.13^b^ ± 0.02	31.23	21.60	32.04	23.54	94.97	84.22	0.53	0.46	62.69	40.83
Chl	10.48^a^ ± 0.04	7.32^b^ ± 0.03	6.63	5.32	7.49	5.62	78.40	89.74	0.83	0.76	12.10	10.39
Fv/Fm	0.75^a^ ± 0.005	0.59^b^ ± 0.004	11.90	9.48	12.68	9.85	88.05	92.57	0.12	0.11	22.99	18.78

FY, forage fresh yield; DY, forage dry weight; A, net photosynthetic rate; gs, stomatal conductance; Tr, transpiration rate; Chl, chlorophyll content; Fv/Fm, photochemical efficiency SE, standard error of the mean. Means with different letter are significantly different in each row, * All genetic variation parameters and heritabilities are significant at 0.05 probability level.

The net photosynthesis rate (A) ranged from 0.96 to 18.22 µmol m^−2^ s^−1^. “Ciakhor” under non-stress conditions in the second year and “GilanTappeh” in the first year and under drought stress treatment had the highest and lowest A, respectively (**Supplementary Table S2**). “Liqvan” (17.42 µmol m^−2^ s^−1^) and “Noqan” (17.25 µmol m^−2^ s^−1^): “Abrumand” (17.25 µmol m^−2^ s^−1^) stood at the second and third rankings for A under non-stress treatment in 2019. The stomatal conductance ranged from 0.01 to 0.38 mol m^−2^ s^−1^. The “Ciakhor” under non-stress conditions in the second year illustrated the highest *g_s_* followed by “Liqvan” (0.37 mol m^−2^ s^−1^) and “Noqan” (0.36 mol m^−2^ s^−1^) in the second year and under a non-stress environment (**Supplementary Table S2**). The “GilanTappeh” (0.23 mol m^−2^ s^−1^) and “Abr2” (4.98 mmol m^−2^ s^−1^) showed the lowest and highest transpiration rate (T_r_) under drought stress conditions in 2018 and non-stress in 2019, respectively. As presented in **Supplementary Table S2**, the chlorophyll content (Chl) varied from 5.75 mg g^−1^ dry weight to 12.02 mg g^−1^ dry weight. Among the assessed accessions, “Sarab” (12.02 mg g^−1^ dry weight) and “Abr2” (12.00 mg g^−1^ dry weight) showed higher Chl content in the second year and under the non-stress condition, whereas the lowest Chl belonged to “GillanTappeh” (5.75 mg g^−1^ dry weight) (**Supplementary Table S2**). Photochemical efficiency (Fv/Fm) ranged from 0.38 in the “Abbasabad” in 2018 under drought stress conditions to 0.85 in the “Ciakhor” in 2019 under non-stress conditions (**Supplementary Table S2**).

The mean for fresh (FY) and dry forage yields (DY) ranged from 332.50 g to 2,026.57 g (FY) and 175.54 g to 1,129.00 g (DY), respectively. “Ciakhor” in 2019 under non-stress and “GilanTappeh” under drought stress conditions in 2018 had the highest and lowest FY and DY, respectively (**Supplementary Table S2**).

### Genetic advance and heritability estimates

The PCV and GCV estimated under non-stress and drought stress treatments are presented in **Table 4**. DY (24.61%, 20.29%) and *g_s_* (47.18%, 41.52%) had the highest PCVs in both irrigation regimes in 2018 and 2019, respectively. FY (22.26%, 18.23%) and A (39.81%, 37.31%) ranked next for PCV under non-stress conditions and drought stress environment in 2018 and 2019, respectively. Chl demonstrated the minimum value for PCV in both years and irrigation regimes (**Table 4**). In 2018, the GCV ranged from 4.37% (Chl) to 19.97% (DY) under non-stress treatment and from 6.63% (Chl) to 46.54% (*g_s_*) under drought treatment. In 2019, DY (19.35%) and FY (17.54%) had the highest value for GCV under non-stress conditions while *g_s_* (39.66%) and A (36.29%) had the highest GCV under drought treatments. The lowest GCV was observed for Chl (4.55%, 5.32%) in two growing seasons (**Table 4**).

The heritability estimates ranged from 65.83% (DY) to 95.37% (T_r_) under non-stress treatment and from 61.22% (FY) to 97.34% (*g_s_*) under drought stress environment in 2018 (**Table 4**). The estimated heritability for *g_s_* ( ${\text{h}}_{b}^{2}$ = 95.21%) under non-stress conditions and T_r_ ( ${\text{h}}_{b}^{2}$ = 94.97%) under drought stress conditions were next in the rankings. In 2019, the heritability of assessed traits ranged from 82.72% for Fv/Fm to 97.94% for T_r_ under non-stress environment and from 84.22% for T_r_ to 94.59% for A under drought stress treatment. The forage yield traits (FY and DY) showed higher heritability in 2019 compared with 2018 under both irrigation regimes (**Table 4**). Furthermore, the photosynthetic traits showed high heritability with low change between two watering regimes across 2 years, while the heritability of FY and DY in 2018 was quite low in both conditions.

The FY in 2018 (251.19) and 2019 (520.23) presented the highest GA, while the lowest GA belonged to *g_s_* (0.07) in 2018 and *g_s_* (0.08) and Fv/Fm (0.08) in 2019 under non-stress treatment. Under drought conditions, the GA ranged from 0.05 for *g_s_* to 113.10 for FY in 2018 and from 0.05 to 269.98 for the same traits in 2019. In 2018, GAM ranged from 8.32% for Chl to 35.63% for FY under non-stress conditions and from 12.10% for Chl to 94.60% for *g_s_* under drought treatment. The DY (38.22%) and *g_s_* (78.05%) demonstrated the highest GAM in 2019 under both irrigation regimes while the lowest GAM was observed in Chl (8.86%, 10.29%) in the same year and irrigation regimes (**Table 4**). The photosynthetic traits showed low GA with low change under non-stress and drought stress conditions over 2 years, while FY and DY represented high GA in both conditions with a significant change over the years (**Table 4**).

### Correlation of traits

**Figure 3:**
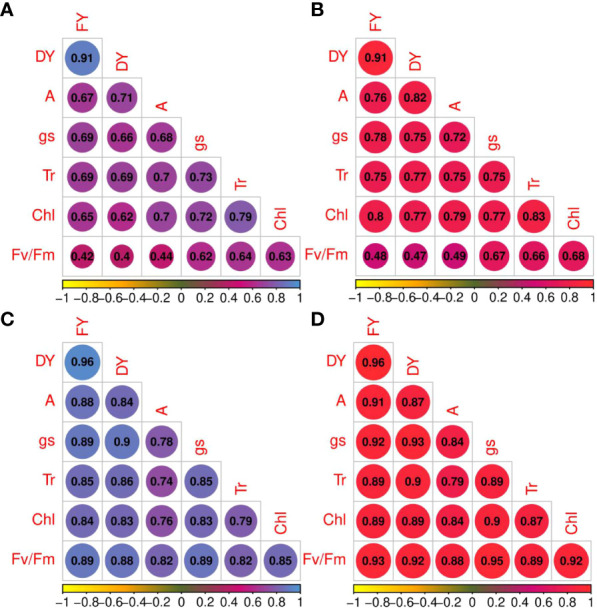
Phenotypic (blue color spectrum) and genotypic (red color spectrum) correlation coefficients for photosynthetic parameters and forage yield traits in 100 *Poa pratensis* accessions evaluated in non-stress **(A, B)** and drought stress **(C, D)** conditions.

The correlation coefficients of forage yield traits and photosynthetic parameters under non-stress and drought stress treatments are shown in **Figure 3**. Under non-stress and drought stress conditions, net photosynthesis rate was strongly correlated with photosynthetic components (r_p_ and r_g_ > 0.70) except with Fv/Fm under normal conditions (**Figure 3**). High correlation coefficients were obtained among other phytochemical traits. For instance, *g_s_* shows strong correlation with T_r_ (r_p_ = 0.73 and r_g_ = 0.75, non-stress; r_p_ = 0.85 and r_g_ = 0.89, drought stress) and Chl (r_p_ = 0.72 and r_g_ = 0.77, non-stress; r_p_ = 0.83 and r_g_ = 0.90, drought stress). T_r_ and Fv/Fm (r_p_ = 0.64 and r_g_ = 0.66, non-stress; r_p_ = 0.82 and r_g_ = 0.89, drought stress) and T_r_ and Chl content showed high correlations. High genotypic and phenotypic (r_p_ = 0.82; r_g_ = 0.88) correlations were obtained between A and Fv/Fm. Additionally, FY was strongly correlated with DY under both irrigation regimes (r_p_ and r_g_ = 0.91, non-stress; r_p_ and r_g_ = 0.96, drought stress). Photosynthetic parameters and forage yield were significantly correlated. Although Fv/Fm had low phenotypic and genotypic correlations with FY and FD under non-stress treatment, they showed strong correlation under drought stress treatment.

Both phenotypic and genotypic correlations of the photosynthetic parameters with FY and DY were higher under drought conditions compared with well-watered control. The results of stepwise regression analysis demonstrated that A, *g_s_*, and T_r_ were the most important contributors to FY (*R* ^2 =^ 64%) and DY (*R* ^2 =^ 67%) variances in well-watered treatment (**Table 5**). Under drought stress conditions, 91% of the FY variation was explained by A, *g_s_*, T_r_, and Fv/Fm. The traits A, *g_s_*, and T_r_ showed high contribution to the total phenotypic variation of DY (**Table 5**).

**Table 5:** Results of stepwise regression analysis between photosynthetic parameters and forage yield traits (FY and DY) in *Poa pratensis* accessions evaluated in non-stress and drought stress conditions.

Treatment	FY					DY				
	Variable entered	Parameter estimate	Partial *R* ^2^	Model *R* ^2^	F value	Variable entered	Parameter estimate	Partial *R* ^2^	Model *R* ^2^	*F* value
Non-stress	*g_s_*	1,641.45	0.5410	0.5410	12.54^***^	A	45.88	0.5841	0.5841	21.09^***^
	A	54.82	0.0775	0.6186	9.42^**^	T_r_	67.37	0.0673	0.6515	7.64^*^
	T_r_	109.43	0.0234	0.6421	6.31^**^	*g_s_*	616.12	0.0193	0.6708	5.64^**^
	Intercept	−470.53	–	–	7.26^**^	Intercept	−460.40	–	–	22.20^***^
Drought stress	*g_s_*	1,134.28	0.8329	0.8329	8.67^**^	*g_s_*	1,312.13	0.8186	0.8186	23.28^***^
	A	31.33	0.0624	0.8953	38.32^***^	T_r_	109.75	0.0368	0.8554	21.32^***^
	Tr	100.85	0.0150	0.9103	13.42^**^	A	13.00	0.0138	0.8692	10.16^**^
	Fv/Fm	279.99	0.0029	0.9132	3.16^*^	Intercept	213.98	–	–	243.23^***^
	Intercept	324.36	–	–	25.55^***^					

n.s., *, **, and *** represent non-significant, significant at p < 0.05, p < 0.01 and p < 0.001, respectively. FY, forage fresh yield; DY, forage dry yield; A, net photosynthetic rate; g_s_, stomatal conductance; T_r_, transpiration rate; Chl, chlorophyll content; Fv/Fm, photochemical efficiency.

### Clustering accessions

**Figure 4:**
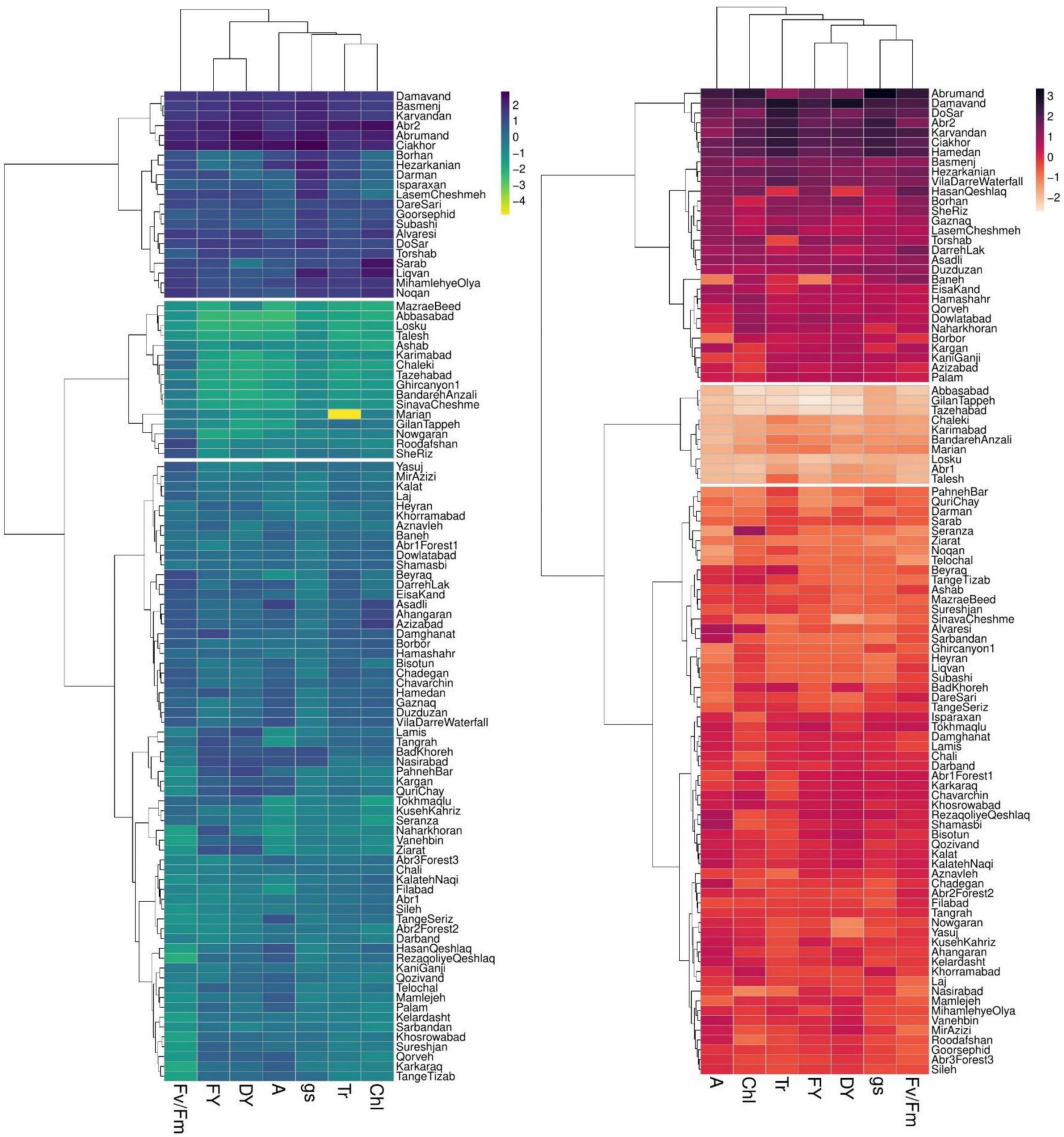
Two-dimensional heatmap dendrogram for 100 P*. pratensis* accessions tested for photosynthetic and forage yield traits under non-stress (blue dendrogram) and drought stress (red dendrogram). Dendrograms illustrate the relation between accessions (rows) and traits (columns) based on variations in color shades. FY, forage fresh yield; DY, forage dry yield; A, net photosynthetic rate; *g_s_*, stomatal conductance; T_r_, transpiration rate; Chl, chlorophyll content; Fv/Fm, photochemical efficiency.

The dendrogram of heatmap clustering of tested *P. pratensis* accessions and evaluated traits under the two irrigation regimes is shown in **Figure 4**. Under non-stress conditions, the accessions were clustered into three distinct groups (**Figure 4**). In group I, the accessions showed high values for all assessed traits. Group II, which comprised 63 accessions, showed relatively moderate values for photosynthetic parameters and forage yield traits. Group III consisted of “MazraeBeed”, “Abbasabad”, “Losku”, “Talesh”, “Ashab”, “Karimabad”, “Chaleki”, “Tazeabad”, “Ghircanyon1”, “BandarehAnzali”, “SinavaCheshme”, “Marian”, “GilanTappeh”, “Nowgaran”, “Roodafshan”, and “SheRiz” accessions had low levels for all measured traits (**Figure 4**).

Under drought stress treatment, the tested *P. pratensis* accessions were divided into three groups (**Figure 4**). Groups I and II represented the highest and lowest values for all measured traits, respectively. Group III comprised 60 accessions with relatively moderate values for forage yield traits and photosynthetic parameters (**Figure 4**). The result of cluster analysis showed that half of the tested accessions belonged to group II under non-stress conditions (**Figure 4**). Eight accessions were placed in group III under drought stress conditions (**Figure 4**) with moderate values for forage yield traits and photosynthetic parameters.

Several accessions placed in the clusters II and III (**Figure 4**) showed low and moderate values for the tested traits under non-stress but high forage yield (FY and DY) under drought stress conditions (**Figure 4**).

## Discussion

Exploiting natural variation from field-collected natural populations can add variation needed to develop new variates. Ecotype variation is the end point of sustained environmental selection, and using these accessions can reveal important and novel variation not available in commercial varieties. Natural variation in underexploited genetic resources such as plant ecotypes is a raw material for the development of new varieties and the continuity of breeding crops for different traits (Flood et al., 2011; Lawson et al., 2012). In the present study, photosynthetic parameters were significantly affected by the moisture regime that was in agreement with results of other studies for the same traits in Kentucky bluegrass (Hu et al., 2010; Hu et al., 2013; Saud et al., 2016; Zhang et al., 2019). The decrease in photosynthetic rate under drought stress is due to the decrease in the supply of water, which decreases the *g_s_* under drought stress to reduce water loss and stomatal closure that then leads to reduced leaf transpiration and an insufficient supply of CO_2_ (Chavers et al., 2009; Ghotbi-Ravandi et al., 2014; Roig-Oliver et al., 2021). Stomatal closure and photosynthesis are the most sensitive events against the adverse effects of drought stress (Quarrie and Jones, 1977; Meng et al., 1999; Xu and Zhou, 2008; Hu et al., 2013; Flexas and Carriqui, 2020). Under drought stress, plants regulate photosynthesis through the balance of water budget by reducing the T_r_, which is an adaptive strategy to avoid the adverse effects of drought (Schreiber et al., 1995; Medrano et al., 2002; Zhang et al., 2022). The photochemical efficiency (Fv/Fm) has been shown as a sensitive indicator of plant photosynthetic performance (Guidi et al., 2019). Reduced Fv/Fm, which is an indicator of the efficiency of excitation energy captured by “open” PSII reaction centers, is associated with downregulation of photosynthesis or decreased photosystem II (PSII) efficiency (Souza et al., 2004; Guidi et al., 2019). The results of our study indicated significant reduction in Fv/Fm quantity under drought stress conditions, which was in line with results of the Fv/Fm ratio in drought compared to the non-stress condition in previous studies of Kentucky bluegrass (Abraham et al., 2008; McCann and Huang, 2008; Hu et al., 2010). Photosynthetic capacity is determined by leaf chlorophyll and photochemical reactions. It has been shown that leaf senescence, which expedites in response to the adverse effects of drought stress, decreases leaf Chl content (Wise and Naylor, 1987). Damage to chlorophyll is almost attributed to damage to membrane, which results in leaf senescence under water-limited conditions (Simon, 1974; Liu and Huang, 2000). In this study, significant reduction was found in Chl content under drought stress compared with the non-stress treatment, which was in line with results of changes in chlorophyll content in Kentucky bluegrass tested under water-limited conditions in the Saud et al. (2016) study. Results of our study showed that *g_s_*, A, and T_r_ had higher reduction under drought compared with the *Chl* content, which shows chlorophyll content and photochemical efficiency that are less sensitive to water-limited conditions than stomatal components in Kentucky bluegrass. It has been shown that early inhibition of photosynthesis under water-limited conditions could be induced by stomatal closure and the possible damage to PSII (Hu et al., 2010). The forage yield of the evaluated *P. pratensis* accessions in this study was significantly decreased under drought stress conditions. The negative impact of drought stress on morphological traits including biomass has been documented in Kentucky bluegrass (Abraham et al., 2008; Liu et al., 2022b).

The particular source of phenotypic variation determines whether the trait has the ability to respond to natural/artificial selections and environmental changes (Byers et al., 2008). Our study showed that the Kentucky bluegrass genotypes had substantial variation for forage yield and several photosynthesis characters over the years and irrigation regimes. However, trait–irrigation treatment interaction was observed in our accessions. The *g_s_* character showed higher phenotypic variation under drought stress conditions compared with normal irrigation treatment. Variation in plant materials is the key prerequisite to successful breeding programs and development of new varieties for use in different environmental conditions. Analysis of heritability and genetic advance helps breeders predict the potential of a population for the improvement of different traits in response to selection. In the current study, the majority of traits including FY, DY, A, *g_s_*, and T_r_ showed moderate to high genetic variability (GCV and PCV), particularly under drought stress treatment, which shows the possibility of the trait improvement through direct and indirect selections. However, the results indicated low variability for Chl and Fv/Fm, suggesting the need for improvement of base population through cross breeding for these traits (Terfa and Gurmu, 2020). The small difference between the GCV and PCV values in our study was consistent with previous studies in different crops (Majidi et al., 2009; Jalata et al., 2011). All the tested traits in this study had relatively high heritability (61.22% to 97.94%), which is critical for successful phenotypic selection. Photosynthetic parameters showed higher heritability compared with forage yield phenotypes in our study. Thus, the association of photosynthesis and forage yield could help use photosynthetic parameters as a criterion for indirect selection for high-yielding varieties under drought stress conditions. Breeding through indirect selection could be more efficient than direct selection in the cases that selection for direct traits is complicated and when indirect traits show high heritability than direct ones (Blum, 2011; Shariatipour et al., 2022). Estimation of the genetic advance (GA) will help to predict selection progress that can be expected as result of exercising selection in a breeder germplasm. High heritability and moderate to high genetic advance were recorded for forage yield and photosynthesis traits except for Chl under non-stress treatment, indicating the predominance of additive gene action for these phenotypes. The use of mean-based genetic advance (GAM) coupled with high heritability helps breeders to better predict the resultant effect of selection for multiple traits compared with selections based on heritability estimates alone. It has been shown that traits with high heritability coupled with moderate genetic advance improved more easily than the traits showing lower GAM and heritability (Singh et al., 2016). The forage-related traits presented higher heritability, GA, and GAM in our population for the non-stress condition compared with drought treatment. According to Blum (2011), yield usually shows higher heritability and greater genetic advance through selection in an optimal environment than in stressed environments. The genotypic variation and the high heritability identified in the current study suggested the higher contribution of genetic components compared with environmental variance in the phenotypic variation of the tested traits. The higher contribution of genetic variance in phenotypic variation accelerates the selection and development of new drought-tolerant varieties with higher forage yield production.

Information on the covariance of traits is useful for predicting how the selection pressure exerted on one trait will result in trade-offs for other traits (Mehri et al., 2009; Kole and Saha, 2013). The correlation of photosynthetic parameters in our study was in agreement with previous studies that have shown co‐regulation of stomatal conductance (*g_s_*) and photosynthesis in plants (Wong et al., 1979; Farquhar et al., 2001; Medrano et al., 2002). The correlation between photosynthetic parameters has been identified in different forage grasses (Fariaszewska et al., 2020; Mastalerczuk et al., 2022). The results of this study indicated that photosynthesis characteristics and forage yield traits were strongly correlated in both irrigation regimes, suggesting the effective role of these parameters in forage production in Kentucky bluegrass. Photosynthesis is the basis of biomass production in plants (Keller et al., 2022). It has been shown that photosynthetic CO_2_ assimilation contributes to approximately 90% of dry matter of crop plants (Lawlor, 1995). In our study, photosynthesis, transpiration, and stomatal conductance had a direct positive effect on the forage yield production, which was in agreement with the results of the Staniak et al. (2018) study in Festulolium [*Festulolium braunii* (K. Richt) A. Camus] and alfalfa (*Medicago* × varia T. Martyn). Results of the Flexas and Carriqui (2020) study have shown that the ratio of *g_m_* (mesophyll conductance) and *g_s_* affects maximizing photosynthesis in plants. In the present study, the identified correlation between photosynthetic characteristics and forage yield phenotypes under drought stress treatment suggests the possibility of successful selection for both high forage yield and higher photosynthesis potential. The result of the interrelation analysis of the tested traits indicated the higher contribution of photosynthetic parameters to the observed variation in forage yield phenotypes (FY and DY) under drought stress environment compared with normal watering treatment, which suggests the critical role of photosynthesis parameters in yield under drought stress conditions (Harbinson and Yin, 2023).

Clustering individuals in a population that provides information about similarities of genotypes helps for selection and crosses between different groups for expanding genetic variation and development of new segregation populations. Results of cluster analysis in our study showed that the assessed accessions were divided into distinct high and low photosynthesis and forage yield groups. The development of a segregating population through crosses between accessions of two high and low productive groups helps in mapping quantitative traits loci and identifying markers associated with traits for use in marker-assisted selection programs in *P. pratensis*.

## Conclusions

The results of our studies showed the significant effects of the watering regime on the photosynthesis system and forge yield traits. Among the tested photosynthetic parameters, stomatal conductance showed a higher correlation with forage yield, which can be suggested as an integrative parameter for identifying drought-tolerant varieties. This work provides supporting information for two research areas. One is the interrelationship of traits and the level of genetic variation for photosynthesis and forage-related traits under two moisture regimes. The other is information on heritability and gain from selection, which shows the potential of our *P. pratensis* population for the improvement of two different sets of traits. The wide variation observed for traits in the ecotypic variation sampled in the accessions tested helps to select good candidates and develop segregating populations through cross-breeding programs to breed drought-tolerant varieties with higher forage yield traits and identify information about QTLs of traits.

## Data availability statement

The original contributions presented in the study are included in the article/**Supplementary Material**. Further inquiries can be directed to the corresponding author.

## Author contributions

NS and ZS performed the experiment, conducted data analysis, and wrote the draft of article; BH conceived and designed the project, reviewed the statistical analyses, and edited the first and final draft of the manuscript; CR contributed to reviewing the analyses and provided critical advice on data analysis. All authors have read and approved the manuscript.
