# Supplementary material for: Genetic variation and response to selection of photosynthetic and forage characteristics in Kentucky bluegrass (Poa pratensis L.) ecotypes under drought conditions
Source: Front Plant Sci. 2023 Nov 10;14:1239860. doi: 10.3389/fpls.2023.1239860 (PMC10667697; doi:10.3389/fpls.2023.1239860)
Supplement: Supplementary file 1 [file Table_1.docx]

| **Supplemental Table S1** Geographic information of original sites of assessed Kentucky bluegrass accessions collected from different part of Iran. | | | | | |
| --- | --- | --- | --- | --- | --- |
| No. | Genotype name | Province | N (Latitude) | E (Longitude) | Altitude |
| 1 | Abbasabad | Mazandaran | 36°43'25.2'' | 51°07'40.4'' | 15 |
| 2 | Abr1Forest1 | Semnan | 36°43'39.5'' | 55°02'29.9'' | 2036 |
| 3 | Abr2Forest2 | Semnan | 36°44'17.7'' | 55°03'07.1'' | 2082 |
| 4 | Abr3Forest3 | Semnan | 36°44'58.5'' | 55°02'32.2'' | 2104 |
| 5 | Abr1 | Semnan | 36°41'14.4'' | 55°07'41.8'' | 1570 |
| 6 | Abr2 | Semnan | 36°42'06.0'' | 55°04'28.7'' | 1736 |
| 7 | Abrumand | Hamedan | 34°54'27.7'' | 48°19'44.5'' | 1739 |
| 8 | Ahangaran | Kermanshah | 34°26'57.6'' | 47°34'29.2'' | 1301 |
| 9 | Alvaresi | Ardabil | 38°11'49.3'' | 47°53'19.0'' | 2923 |
| 10 | Asadli | North Khorasan | 37°17'57.2'' | 57°21'54.8'' | 1757 |
| 11 | Ashab | Kurdistan | 35°41'24.4'' | 47°06'34.5'' | 2023 |
| 12 | Azizabad | Kermanshah | 34°31'31.0'' | 48°00'48.3'' | 1485 |
| 13 | Aznãvleh | Isfahan | 33°07'51.7'' | 50°06'48.5'' | 2447 |
| 14 | BadKhoreh | Hamedan | 34°45'02.8'' | 48°05'30.5'' | 1566 |
| 15 | BandarehAnzali | Gilan | 37°27'37.2'' | 49°34'27.0'' | 8 |
| 16 | Baneh | Kurdistan | 36°01'10.4'' | 45°55'43.2'' | 1596 |
| 17 | Basmenj | East Azerbaijan | 37°59'15.2'' | 46°28'36.5'' | 1723 |
| 18 | Beyraq | East Azerbaijan | 37°53'13.4'' | 46°27'57.9'' | 1939 |
| 19 | Bisotun | Kermanshah | 34°22'52.7'' | 47°26'26.0'' | 1313 |
| 20 | Borbor | West Azerbaijan | 37°37'34.5'' | 45°07'08.9'' | 1306 |
| 21 | Borhan | West Azerbaijan | 36°41'26.0'' | 45°57'54.1'' | 1584 |
| 22 | Chadegan | Isfahan | 32°46'42.6'' | 50°39'09.7'' | 2133 |
| 23 | Chaleki | Golestan | 36°49'56.9'' | 54°17'45.5'' | 50 |
| 24 | Chali | Kurdistan | 36°07'26.6'' | 46°48'18.6'' | 2129 |
| 25 | Chavarchin | West Azerbaijan | 36°34'45.2'' | 46°04'43.7'' | 1420 |
| 26 | Ciakhor | Kermanshah | 34°07'30.9'' | 46°36'24.2 | 1387 |
| 27 | Damavand | Tehran | 35°40'44.6'' | 52°03'48.7'' | 1893 |
| 28 | Damghanat | Fars | 30°05'41.8'' | 52°05'51.2'' | 2157 |
| 29 | Darband | Lorestan | 33°26'24.1'' | 49°19'17.2'' | 1764 |
| 30 | DareSari | Isfahan | 33°14'21.8'' | 49°58'43.2'' | 2223 |
| 31 | Darman | West Azerbaijan | 36°44'37.8'' | 45°53'16.4'' | 1773 |
| 32 | DarrehLak | West Azerbaijan | 36°54'41.7'' | 45°45'00.7'' | 1271 |
| 33 | DoSar | Kurdistan | 35°05'18.3'' | 48°02'03.6'' | 1821 |
| 34 | Dowlatabad | North Khorasan | 36°57'57.6'' | 57°32'27.6'' | 1265 |
| 35 | Duzduzan | East Azerbaijan | 37°56'39.0'' | 47°07'55.7'' | 1676 |
| 36 | EisaKand | West Azerbaijan | 36°39'45.7'' | 45°59'43.6'' | 1498 |
| 37 | Filabad | Chaharmahal and Bakhtiari | 32°17'09.5'' | 50°31'24.5'' | 2082 |
| 38 | Gaznaq | West Azerbaijan | 37°41'20.6'' | 45°11'53.2'' | 1653 |
| 39 | Ghircanyon1 | Ilam | 33°46'26.6'' | 46°40'53.6'' | 1045 |
| 40 | GilanTappeh | Golestan | 37°19'45.9'' | 55°28'57.6'' | 154 |
| 41 | Goorsephid | Tehran | 35°44'14.4'' | 53°02'33.5'' | 2351 |
| 42 | Hamashahr | Fars | 30°15'32.4'' | 52°16'16.5'' | 1893 |
| 43 | Hamedan | Hamedan | 34°49'53.3'' | 48°29'55.8'' | 1788 |
| 44 | HasanQeshlaq | Hamedan | 35°02'24.6'' | 48°19'0.9'' | 1851 |
| 45 | Heyran | Gilan | 38°23'59.0'' | 48°36'50.6'' | 782 |
| 46 | Hezarkanian | Kurdistan | 35°44'59.8'' | 47°05'11.7'' | 1882 |
| 47 | Isparaxan | East Azerbaijan | 37°48'54.1'' | 46°23'55.1'' | 2497 |
| 48 | Kalat | North Khorasan | 37°13'47.0'' | 57°23'24.3'' | 1596 |
| 49 | KalatehNaqi | North Khorasan | 37°23'32.3'' | 57°17'9.1'' | 1242 |
| 50 | KaniGanji | Kurdistan | 35°13'04.7'' | 47°37'55.8'' | 1874 |
| 51 | Kargan | East Azerbaijan | 37°53'01.2'' | 46°43'58.8'' | 1888 |
| 52 | Karimabad | Mazandaran | 36°49'57.0'' | 50°50'24.9'' | 12 |
| 53 | Karkaraq | Ardabil | 38°17'57.9'' | 48°20'50.4'' | 1331 |
| 54 | Karvandan | Kurdistan | 35°18'38.9'' | 47°21'18.0'' | 1843 |
| 55 | Kelardasht | Mazandaran | 36°29'45.0'' | 51°08'42.7'' | 1299 |
| 56 | Khorramabad | Lorestan | 33°25'54.4'' | 48°17'14.9'' | 1157 |
| 57 | Khosrowabad | Hamedan | 34°38'02.8'' | 48°02'54.4'' | 1505 |
| 58 | KusehKahriz | West Azerbaijan | 36°55'59.8'' | 45°40'17.0'' | 1262 |
| 59 | Laj | West Azerbaijan | 36°54'12.7'' | 45°47'37.2'' | 1286 |
| 60 | Lamis | Lorestan | 33°25'33.1'' | 49°30'23.3'' | 1884 |
| 61 | LasemCheshmeh | Tehran | 35°44'05.8'' | 52°41'59.3'' | 1903 |
| 62 | Liqvan | East Azerbaijan | 37°49'33.3'' | 46°25'35.5'' | 2215 |
| 63 | Losku | Gilan | 37°19'25.5'' | 49°56'13.3'' | 11 |
| 64 | Mamlejeh | North Khorasan | 37°29'50.3'' | 57°11'23.3 | 1322 |
| 65 | Marian | Gilan | 37°48'29.9'' | 48°49'37.1'' | 174 |
| 66 | MazraeBeed | Hamedan | 34°45'08.9'' | 48°05'53.9'' | 2031 |
| 67 | MihamlehyeOlya | Hamedan | 35°07'24.4'' | 48°17'01.6'' | 2132 |
| 68 | MirAzizi | Kermanshah | 34°27'55.6'' | 47°36'24.4 | 1310 |
| 69 | Naharkhoran | Golestan | 36°45'46.5'' | 54°28'32.4'' | 516 |
| 70 | Nasirabad | Chaharmahal and Bakhtiari | 32°22'42.8'' | 50°19'23.2'' | 2431 |
| 71 | Noqan | Isfahan | 33°11'01.4'' | 50°04'02.8'' | 2242 |
| 72 | Nowgaran | Kurdistan | 35°27'43.9'' | 47°12'28.4'' | 2050 |
| 73 | PahnehBar | Hamedan | 34°56'40.2'' | 48°16'18.8'' | 1880 |
| 74 | Palam | Gilan | 36°55'35.6'' | 50°15'21.1'' | 334 |
| 75 | Qorveh | Kurdistan | 35°11'16.5'' | 47°44'53.8'' | 1920 |
| 76 | Qozivand | Kermanshah | 34°24'48.5'' | 47°27'53.9'' | 1290 |
| 77 | QuriChay | Kurdistan | 35°23'02.5'' | 47°18'16.8'' | 1875 |
| 78 | RezaqoliyeQeshlaq | Ardabil | 38°06'40.8'' | 48°08'31.0'' | 1482 |
| 79 | Roodafshan | Tehran | 35°39'48.1'' | 52°28'07.2'' | 2157 |
| 80 | Sarab | East Azerbaijan | 37°56'08.6'' | 47°28'59.0'' | 1700 |
| 81 | Sarbandan | Tehran | 35°38'06.7'' | 52°18'50.3'' | 2178 |
| 82 | Seranza | Tehran | 35°45'15.4'' | 52°54'24.1'' | 2230 |
| 83 | Shamasbi | Ardabil | 38°11'04.7'' | 48°13'31.9'' | 1417 |
| 84 | TangeSehRiz1 | Chaharmahal and Bakhtiari | 31°25'58.4'' | 51°09'24.5'' | 1983 |
| 85 | Sileh | Mazandaran | 36°36'19.3'' | 51°23'01.0'' | 144 |
| 86 | SinavaCheshme | Hamedan | 35°09'24.3'' | 48°15'46.0'' | 2266 |
| 87 | Subashi | Chaharmahal and Bakhtiari | 32°18'57.3'' | 50°39'48.3'' | 2076 |
| 88 | Sureshjan | Gilan | 37°47'38.0'' | 48°53'35.8'' | 102 |
| 89 | Talesh | Kohgiluyeh and BoyerAhmad | 30°44'47.3'' | 51°28'39.8'' | 1840 |
| 90 | TangeSehRiz2 | Kohgiluyeh and BoyerAhmad | 30°44'41.8'' | 51°28'50.7'' | 1710 |
| 91 | TangeTizab | Fars | 30°27'37.4'' | 51°43'04.4'' | 2158 |
| 92 | Tangrah | Golestan | 37°23'55.9'' | 55°48'08.5'' | 493 |
| 93 | Tazehabad | Mazandaran | 36°41'53.9'' | 53°36'33.2'' | 73 |
| 94 | Telochal | Mazandaran | 36°32'00.1'' | 51°09'59.1'' | 1098 |
| 95 | Tokhmaqlu | Isfahan | 33°11'56.7'' | 50°06'08.5'' | 2318 |
| 96 | Torshab | Lorestan | 33°28'13.9'' | 49°11'29.3'' | 1626 |
| 97 | Vanehbin | Gilan | 38°25'29.6'' | 48°34'55.1'' | 1318 |
| 98 | Vila Darre Waterfall | Ardabil | 38°10'37.1'' | 48°03'26.7'' | 1787 |
| 99 | Yasuj | Kohgiluyeh and BoyerAhmad | 30°40'30.5'' | 51°37'14.5'' | 2010 |
| 100 | Ziarat | Golestan | 36°41'18.6'' | 54°27'58.2'' | 1108 |
