# Supplementary material for: Genetic variation and response to selection of photosynthetic and forage characteristics in Kentucky bluegrass (Poa pratensis L.) ecotypes under drought conditions
Source: Front Plant Sci. 2023 Nov 10;14:1239860. doi: 10.3389/fpls.2023.1239860 (PMC10667697; doi:10.3389/fpls.2023.1239860)
Supplement: Supplementary file 2 [file Table_2.docx]

| **Supplemental Table S2** Mean values of photosynthetic characters and forage yield traits measured from one-hundred accessions of Kentucky Bluegrass evaluated in non-stress and drought stress environments during years 2018 and 2019. | | | | | | | | | | |
| --- | --- | --- | --- | --- | --- | --- | --- | --- | --- | --- |
| G | Non-stress | | | | |  | Drought stress | | | |
|  | 2018 | | 2019 | | |  | 2018 | | 2019 | |
|  | FY | ± SE | FY | | ± SE |  | FY | ± SE | FY | ± SE |
| Abbasabad | 488.40 | ± 6.37 | 920.01 | | ± 9.47 |  | 346.00 | ± 6.00 | 672.91 | ± 5.99 |
| Abr1 | 645.50 | ± 14.66 | 1264.50 | | ± 14.17 |  | 351.56 | ± 7.40 | 796.08 | ± 3.02 |
| Abr1Forest1 | 653.95 | ± 0.95 | 1315.70 | | ± 7.59 |  | 500.69 | ± 4.31 | 1084.38 | ± 8.62 |
| Abr2 | 1488.04 | ± 515.63 | 1472.10 | | ± 512.34 |  | 611.07 | ± 4.34 | 1252.75 | ± 7.75 |
| Abr2Forest2 | 628.21 | ± 7.25 | 1243.82 | | ± 42.02 |  | 440.95 | ± 3.64 | 1024.61 | ± 17.44 |
| Abr3Forest3 | 631.93 | ± 0.27 | 1272.75 | | ± 15.11 |  | 465.89 | ± 5.15 | 989.82 | ± 2.85 |
| Abrumand | 950.58 | ± 6.53 | 1927.23 | | ± 6.58 |  | 911.53 | ± 383.97 | 958.06 | ± 370.88 |
| Ahangaran | 734.30 | ± 2.30 | 1490.86 | | ± 16.71 |  | 471.68 | ± 3.45 | 1015.68 | ± 0.26 |
| Alvaresi | 701.66 | ± 1.09 | 1898.72 | | ± 2.46 |  | 444.15 | ± 6.17 | 979.52 | ± 0.44 |
| Asadli | 733.65 | ± 2.45 | 1490.14 | | ± 4.85 |  | 594.10 | ± 1.10 | 1121.43 | ± 1.57 |
| Ashab | 449.46 | ± 7.5 | 1314.69 | | ± 11.64 |  | 422.50 | ± 7.55 | 1016.43 | ± 6.54 |
| Azizabad | 760.82 | ± 3.18 | 1528.29 | | ± 13.70 |  | 509.46 | ± 0.03 | 1089.21 | ± 2.96 |
| Aznãvleh | 630.42 | ± 3.93 | 1708.69 | | ± 1.72 |  | 423.20 | ± 2.48 | 1122.19 | ± 6.03 |
| BadKhoreh | 861.98 | ± 0.59 | 1663.56 | | ± 2.96 |  | 502.77 | ± 8.71 | 904.88 | ± 2.12 |
| BandarehAnzali | 518.59 | ± 1.71 | 1092.17 | | ± 32.96 |  | 396.88 | ± 18.15 | 880.06 | ± 7.96 |
| Baneh | 849.56 | ± 7.56 | 1315.76 | | ± 10.26 |  | 520.37 | ± 0.73 | 783.50 | ± 5.50 |
| Basmenj | 894.96 | ± 6.04 | 1886.19 | | ± 4.01 |  | 572.22 | ± 13.34 | 1229.92 | ± 4.08 |
| Beyraq | 656.11 | ± 4.64 | 1629.88 | | ± 1.52 |  | 435.31 | ± 0.49 | 941.11 | ± 0.52 |
| Bisotun | 732.11 | ± 6.07 | 1370.88 | | ± 7.09 |  | 494.66 | ± 8.13 | 1103.32 | ± 2.32 |
| Borbor | 730.16 | ± 8.36 | 1389.11 | | ± 15.19 |  | 503.81 | ± 5.84 | 1109.05 | ± 13.95 |
| Borhan | 882.64 | ± 2.36 | 1298.87 | | ± 7.76 |  | 532.47 | ± 0.18 | 1234.05 | ± 9.95 |
| Chadegan | 759.03 | ± 39.43 | 1418.74 | | ± 1.29 |  | 465.60 | ± 0.70 | 1016.67 | ± 3.56 |
| Chaleki | 495.70 | ± 6.35 | 1090.64 | | ± 32.30 |  | 406.53 | ± 3.23 | 837.20 | ± 8.54 |
| Chali | 738.47 | ± 0.59 | 1269.93 | | ± 18.52 |  | 486.00 | ± 10.00 | 1073.00 | ± 2.00 |
| Chavarchin | 735.43 | ± 4.14 | 1398.69 | | ± 4.53 |  | 512.49 | ± 5.51 | 1082.60 | ± 5.60 |
| Ciakhor | 969.40 | ± 1.91 | 2026.57 | | ± 9.15 |  | 627.66 | ± 10.02 | 1290.58 | ± 0.80 |
| Damavand | 890.67 | ± 4.33 | 1864.01 | | ± 4.00 |  | 646.01 | ± 3.65 | 1339.80 | ± 1.80 |
| Damghanat | 807.16 | ± 5.78 | 1792.07 | | ± 0.40 |  | 494.61 | ± 8.39 | 1055.70 | ± 2.10 |
| Darband | 624.67 | ± 7.33 | 1282.74 | | ± 1.27 |  | 529.98 | ± 11.37 | 970.75 | ± 2.85 |
| DareSari | 652.68 | ± 7.91 | 1852.43 | | ± 12.39 |  | 440.71 | ± 4.64 | 956.23 | ± 9.51 |
| Darman | 878.38 | ± 1.92 | 1712.59 | | ± 4.56 |  | 460.89 | ± 4.61 | 849.72 | ± 38.72 |
| DarrehLak | 708.28 | ± 4.71 | 1437.70 | | ± 16.20 |  | 595.46 | ± 6.20 | 1114.32 | ± 3.59 |
| DoSar | 882.27 | ± 1.74 | 1839.89 | | ± 12.27 |  | 610.97 | ± 3.39 | 1290.43 | ± 6.57 |
| Dowlatabad | 842.96 | ± 2.04 | 1347.84 | | ± 1.52 |  | 538.43 | ± 15.67 | 1186.10 | ± 4.90 |
| Duzduzan | 686.79 | ± 4.21 | 1392.33 | | ± 5.81 |  | 552.24 | ± 17.75 | 1177.53 | ± 13.53 |
| EisaKand | 772.29 | ± 12.78 | 1566.29 | | ± 9.11 |  | 525.58 | ± 10.99 | 1128.93 | ± 1.51 |
| Filabad | 633.79 | ± 0.31 | 1272.39 | | ± 9.39 |  | 456.22 | ± 0.37 | 1013.97 | ± 6.10 |
| Gaznaq | 682.05 | ± 13.95 | 1358.42 | | ± 16.2 |  | 539.00 | ± 8.00 | 1178.94 | ± 6.94 |
| Ghircanyon1 | 496.73 | ± 29.07 | 1102.61 | | ± 1.65 |  | 431.52 | ± 0.58 | 933.59 | ± 8.31 |
| GilanTappeh | 523.29 | ± 5.64 | 1301.91 | | ± 6.54 |  | 332.50 | ± 2.50 | 646.00 | ± 4.00 |
| Goorsephid | 634.77 | ± 13.06 | 1851.06 | | ± 37.34 |  | 431.30 | ± 0.70 | 1057.44 | ± 4.56 |
| Hamashahr | 788.40 | ± 9.65 | 1501.70 | | ± 3.84 |  | 519.07 | ± 6.93 | 1129.21 | ± 13.79 |
| Hamedan | 677.94 | ± 0.71 | 1793.80 | | ± 0.29 |  | 615.69 | ± 9.71 | 1253.69 | ± 1.46 |
| HasanQeshlaq | 853.23 | ± 2.77 | 1239.47 | | ± 1.82 |  | 587.08 | ± 12.93 | 1211.04 | ± 2.04 |
| Heyran | 588.14 | ± 4.68 | 1729.17 | | ± 1.49 |  | 430.08 | ± 2.07 | 934.82 | ± 8.16 |
| Hezarkanian | 910.69 | ± 3.31 | 1243.79 | | ± 4.56 |  | 586.07 | ± 18.17 | 1220.45 | ± 1.52 |
| Isparaxan | 602.06 | ± 3.60 | 1829.84 | | ± 12.6 |  | 538.24 | ± 2.24 | 1008.60 | ± 4.40 |
| Kalat | 740.56 | ± 0.40 | 1381.70 | | ± 2.34 |  | 495.00 | ± 3.00 | 1061.83 | ± 3.83 |
| KalatehNaqi | 692.16 | ± 7.90 | 1376.12 | | ± 14.43 |  | 488.01 | ± 0.55 | 1067.71 | ± 9.29 |
| KaniGanji | 628.36 | ± 5.45 | 1390.08 | | ± 4.56 |  | 528.78 | ± 12.38 | 1138.73 | ± 2.79 |
| Kargan | 805.90 | ± 6.08 | 1568.27 | | ± 5.35 |  | 526.78 | ± 12.91 | 1114.41 | ± 4.41 |
| Karimabad | 552.80 | ± 11.10 | 1117.49 | | ± 12.07 |  | 378.46 | ± 2.54 | 866.54 | ± 10.11 |
| Karkaraq | 746.81 | ± 0.00 | 1466.81 | | ± 5.82 |  | 485.95 | ± 6.95 | 1080.00 | ± 3.00 |
| Karvandan | 889.86 | ± 4.86 | 1904.96 | | ± 10.63 |  | 613.62 | ± 8.44 | 1316.61 | ± 1.39 |
| Kelardasht | 731.74 | ± 4.41 | 1425.71 | | ± 10.28 |  | 457.66 | ± 17.65 | 1048.29 | ± 4.34 |
| Khorramabad | 580.44 | ± 22.65 | 1644.32 | | ± 11.92 |  | 440.02 | ± 1.13 | 1009.73 | ± 4.64 |
| Khosrowabad | 770.12 | ± 23.84 | 1491.61 | | ± 2.52 |  | 503.53 | ± 8.47 | 1080.57 | ± 7.43 |
| KusehKahriz | 817.55 | ± 13.03 | 1239.06 | | ± 6.25 |  | 506.81 | ± 13.19 | 1070.72 | ± 0.26 |
| Laj | 729.64 | ± 6.60 | 1434.48 | | ± 6.55 |  | 451.05 | ± 10.49 | 1025.91 | ± 14.82 |
| Lamis | 846.40 | ± 9.60 | 1593.12 | | ± 1.52 |  | 489.25 | ± 1.25 | 1067.83 | ± 4.17 |
| LasemCheshmeh | 890.81 | ± 8.91 | 1744.56 | | ± 9.11 |  | 525.77 | ± 15.75 | 1116.63 | ± 8.37 |
| Liqvan | 580.17 | ± 25.89 | 1960.25 | | ± 5.02 |  | 441.28 | ± 7.04 | 940.79 | ± 10.69 |
| Losku | 495.40 | ± 3.00 | 968.10 | | ± 15.30 |  | 404.00 | ± 6.00 | 697.72 | ± 23.88 |
| Mamlejeh | 732.38 | ± 7.55 | 1381.23 | | ± 6.86 |  | 484.75 | ± 4.75 | 1058.57 | ± 3.57 |
| Marian | 539.27 | ± 14.86 | 1406.06 | | ± 9.09 |  | 409.62 | ± 6.99 | 915.83 | ± 2.31 |
| MazraeBeed | 480.00 | ± 5.96 | 990.09 | | ± 6.24 |  | 449.96 | ± 4.00 | 1053.50 | ± 2.50 |
| MihamlehyeOlya | 632.51 | ± 3.24 | 1893.93 | | ± 2.02 |  | 489.00 | ± 8.00 | 957.97 | ± 5.03 |
| MirAzizi | 728.78 | ± 4.76 | 1330.11 | | ± 1.29 |  | 479.02 | ± 0.49 | 1051.12 | ± 6.88 |
| Naharkhoran | 695.87 | ± 9.51 | 1802.50 | | ± 5.50 |  | 567.82 | ± 7.58 | 1095.74 | ± 3.98 |
| Nasirabad | 689.34 | ± 7.45 | 1879.98 | | ± 0.69 |  | 427.86 | ± 4.21 | 1105.84 | ± 1.36 |
| Noqan | 693.39 | ± 3.81 | 1906.60 | | ± 9.73 |  | 428.10 | ± 1.19 | 921.12 | ± 0.75 |
| Nowgaran | 562.04 | ± 3.96 | 1014.56 | | ± 5.44 |  | 453.55 | ± 13.66 | 997.68 | ± 2.32 |
| PahnehBar | 854.44 | ± 6.56 | 1609.32 | | ± 5.57 |  | 508.79 | ± 8.79 | 753.12 | ± 8.30 |
| Palam | 600.16 | ± 3.00 | 1697.91 | | ± 1.01 |  | 534.83 | ± 14.34 | 1093.57 | ± 2.02 |
| Qorveh | 804.37 | ± 7.94 | 1550.82 | | ± 1.59 |  | 528.26 | ± 14.14 | 1126.97 | ± 6.03 |
| Qozivand | 627.62 | ± 4.25 | 1422.52 | | ± 15.19 |  | 479.95 | ± 4.59 | 1049.92 | ± 3.65 |
| QuriChay | 843.82 | ± 6.52 | 1598.06 | | ± 2.41 |  | 547.09 | ± 19.99 | 711.10 | ± 2.08 |
| RezaqoliyeQeshlaq | 757.42 | ± 8.58 | 1479.22 | | ± 2.02 |  | 518.53 | ± 18.25 | 1094.65 | ± 9.35 |
| Roodafshan | 612.04 | ± 5.53 | 1207.74 | | ± 7.97 |  | 475.22 | ± 6.83 | 1018.45 | ± 2.08 |
| Sarab | 872.62 | ± 0.19 | 1694.95 | | ± 1.95 |  | 532.84 | ± 6.44 | 907.50 | ± 6.50 |
| Sarbandan | 587.33 | ± 25.6 | 1580.78 | | ± 1.26 |  | 424.00 | ± 4.00 | 916.33 | ± 1.23 |
| Seranza | 544.13 | ± 3.87 | 1611.58 | | ± 11.37 |  | 432.00 | ± 6.67 | 913.61 | ± 0.31 |
| Shamasbi | 749.44 | ± 4.56 | 1448.17 | | ± 3.38 |  | 490.59 | ± 6.71 | 1061.61 | ± 17.61 |
| TangeSehRiz1 | 599.24 | ± 14.24 | 1235.94 | | ± 8.67 |  | 537.38 | ± 6.63 | 1202.12 | ± 1.71 |
| Sileh | 631.96 | ± 4.69 | 1311.81 | | ± 12.81 |  | 453.07 | ± 13.67 | 1028.06 | ± 14.77 |
| SinavaCheshme | 489.58 | ± 13.90 | 1128.50 | | ± 2.50 |  | 406.91 | ± 6.65 | 986.93 | ± 10.25 |
| Subashi | 693.87 | ± 1.74 | 1842.27 | | ± 3.24 |  | 439.85 | ± 6.60 | 938.10 | ± 11.44 |
| Sureshjan | 624.94 | ± 4.68 | 1713.19 | | ± 13.93 |  | 452.44 | ± 2.31 | 943.95 | ± 18.34 |
| Talesh | 505.96 | ± 4.56 | 1053.50 | | ± 4.50 |  | 357.50 | ± 6.50 | 827.44 | ± 0.21 |
| TangeSehRiz2 | 606.02 | ± 23.83 | 1287.91 | | ± 2.91 |  | 546.86 | ± 22.76 | 872.55 | ± 11.55 |
| TangeTizab | 615.84 | ± 4.16 | 1659.28 | | ± 13.16 |  | 445.01 | ± 10.64 | 934.31 | ± 3.49 |
| Tangrah | 762.37 | ± 17.42 | 1785.54 | | ± 0.46 |  | 487.93 | ± 11.51 | 1002.77 | ± 1.29 |
| Tazehabad | 514.09 | ± 1.82 | 1082.13 | | ± 6.57 |  | 362.54 | ± 4.46 | 669.02 | ± 2.99 |
| Telochal | 586.80 | ± 7.69 | 1785.46 | | ± 7.54 |  | 433.47 | ± 8.73 | 906.81 | ± 2.35 |
| Tokhmaqlu | 541.84 | ± 18.31 | 1801.53 | | ± 4.60 |  | 532.58 | ± 9.67 | 1088.68 | ± 1.59 |
| Torshab | 891.56 | ± 5.44 | 1774.83 | | ± 12.18 |  | 564.90 | ± 7.97 | 1191.26 | ± 1.74 |
| Vanehbin | 728.42 | ± 8.47 | 1569.83 | | ± 14.68 |  | 476.45 | ± 1.01 | 1050.82 | ± 6.65 |
| Vila Darre Waterfall | 689.36 | ± 4.64 | 1474.98 | | ± 5.18 |  | 579.34 | ± 2.63 | 1235.67 | ± 0.75 |
| Yasuj | 864.43 | ± 4.58 | 1121.95 | | ± 5.39 |  | 453.41 | ± 14.51 | 1026.65 | ± 12.28 |
| Ziarat | 677.15 | ± 5.89 | 1804.90 | | ± 11.01 |  | 418.48 | ± 6.07 | 890.23 | ± 7.29 |
| G | Non-stress | | | | |  | Drought stress | | | |
|  | 2018 | | 2019 | | |  | 2018 | | 2019 | |
|  | DY | ± SE | DY | | ± SE |  | DY | ± SE | DY | ± SE |
| Abbasabad | 234.00 | ± 1.00 | 456.00 | | ± 2.00 |  | 201.94 | ± 3.18 | 380.88 | ± 9.38 |
| Abr1 | 351.86 | ± 5.93 | 693.50 | | ± 3.50 |  | 258.33 | ± 2.18 | 431.58 | ± 4.16 |
| Abr1Forest1 | 391.88 | ± 3.44 | 769.00 | | ± 1.00 |  | 263.02 | ± 3.20 | 687.70 | ± 15.25 |
| Abr2 | 783.28 | ± 31.37 | 820.38 | | ± 31.35 |  | 381.90 | ± 0.50 | 775.50 | ± 3.95 |
| Abr2Forest2 | 318.45 | ± 4.89 | 733.00 | | ± 13.00 |  | 237.76 | ± 2.96 | 598.64 | ± 13.72 |
| Abr3Forest3 | 360.05 | ± 1.81 | 743.00 | | ± 4.00 |  | 309.57 | ± 4.15 | 568.30 | ± 1.47 |
| Abrumand | 611.44 | ± 2.83 | 1082.50 | | ± 1.50 |  | 518.83 | ± 269.65 | 602.49 | ± 267.33 |
| Ahangaran | 335.72 | ± 5.70 | 841.00 | | ± 13.00 |  | 289.77 | ± 4.33 | 630.54 | ± 9.10 |
| Alvaresi | 372.56 | ± 10.56 | 1086.50 | | ± 29.50 |  | 241.70 | ± 14.46 | 566.16 | ± 2.39 |
| Asadli | 343.43 | ± 4.81 | 843.50 | | ± 3.50 |  | 334.96 | ± 2.21 | 680.09 | ± 0.16 |
| Ashab | 258.93 | ± 7.00 | 762.50 | | ± 4.50 |  | 196.00 | ± 4.79 | 595.39 | ± 5.95 |
| Azizabad | 345.45 | ± 8.55 | 860.50 | | ± 2.50 |  | 283.49 | ± 0.33 | 693.96 | ± 8.78 |
| Aznãvleh | 313.62 | ± 9.03 | 724.00 | | ± 2.00 |  | 222.22 | ± 0.45 | 667.14 | ± 11.77 |
| BadKhoreh | 347.39 | ± 4.39 | 966.50 | | ± 0.50 |  | 283.85 | ± 13.8 | 649.90 | ± 4.52 |
| BandarehAnzali | 256.57 | ± 4.71 | 522.50 | | ± 7.50 |  | 225.13 | ± 10.72 | 488.64 | ± 7.55 |
| Baneh | 347.61 | ± 8.39 | 705.00 | | ± 2.00 |  | 268.10 | ± 4.74 | 670.35 | ± 1.27 |
| Basmenj | 529.89 | ± 2.00 | 1043.50 | | ± 1.50 |  | 332.74 | ± 0.40 | 764.12 | ± 3.32 |
| Beyraq | 363.50 | ± 9.50 | 768.00 | | ± 6.00 |  | 236.93 | ± 5.01 | 530.73 | ± 0.12 |
| Bisotun | 335.13 | ± 7.83 | 781.50 | | ± 3.50 |  | 281.27 | ± 11.23 | 703.73 | ± 0.41 |
| Borbor | 334.61 | ± 8.64 | 788.50 | | ± 6.50 |  | 282.68 | ± 0.85 | 711.68 | ± 5.89 |
| Borhan | 377.05 | ± 6.95 | 782.00 | | ± 1.00 |  | 315.85 | ± 4.57 | 775.37 | ± 7.66 |
| Chadegan | 327.92 | ± 6.30 | 792.50 | | ± 0.50 |  | 274.50 | ± 2.78 | 597.29 | ± 6.42 |
| Chaleki | 259.64 | ± 1.45 | 532.50 | | ± 19.50 |  | 213.60 | ± 1.22 | 449.56 | ± 7.38 |
| Chali | 372.39 | ± 3.03 | 744.50 | | ± 5.50 |  | 259.38 | ± 5.74 | 668.71 | ± 2.79 |
| Chavarchin | 338.56 | ± 6.8 | 788.50 | | ± 4.50 |  | 261.93 | ± 0.21 | 694.62 | ± 1.84 |
| Ciakhor | 541.28 | ± 8.28 | 1129.00 | | ± 3.00 |  | 375.84 | ± 7.35 | 801.67 | ± 3.45 |
| Damavand | 486.16 | ± 1.97 | 1029.50 | | ± 6.50 |  | 461.94 | ± 8.46 | 864.95 | ± 4.00 |
| Damghanat | 295.89 | ± 5.47 | 914.50 | | ± 3.50 |  | 254.08 | ± 4.48 | 667.49 | ± 1.01 |
| Darband | 325.58 | ± 8.58 | 737.50 | | ± 6.50 |  | 310.48 | ± 7.49 | 611.80 | ± 4.10 |
| DareSari | 351.78 | ± 3.42 | 1011.00 | | ± 17.00 |  | 232.25 | ± 2.31 | 539.70 | ± 1.34 |
| Darman | 354.86 | ± 5.71 | 815.00 | | ± 6.00 |  | 272.70 | ± 6.28 | 564.89 | ± 8.99 |
| DarrehLak | 486.22 | ± 1.78 | 814.50 | | ± 9.50 |  | 389.10 | ± 1.90 | 563.87 | ± 2.20 |
| DoSar | 417.31 | ± 15.37 | 1025.50 | | ± 5.50 |  | 281.37 | ± 2.59 | 830.22 | ± 0.38 |
| Dowlatabad | 398.15 | ± 6.15 | 747.00 | | ± 4.00 |  | 311.75 | ± 2.19 | 709.14 | ± 0.95 |
| Duzduzan | 367.12 | ± 0.88 | 816.50 | | ± 2.50 |  | 337.79 | ± 5.00 | 728.43 | ± 6.37 |
| EisaKand | 344.75 | ± 1.33 | 916.00 | | ± 10.00 |  | 318.44 | ± 10.96 | 660.85 | ± 0.89 |
| Filabad | 310.68 | ± 2.42 | 743.50 | | ± 0.50 |  | 259.53 | ± 2.49 | 599.08 | ± 7.10 |
| Gaznaq | 357.91 | ± 4.09 | 764.50 | | ± 2.50 |  | 319.56 | ± 9.70 | 635.85 | ± 14.64 |
| Ghircanyon1 | 250.14 | ± 9.55 | 547.50 | | ± 0.50 |  | 230.27 | ± 3.43 | 545.26 | ± 24.53 |
| GilanTappeh | 248.27 | ± 1.32 | 569.50 | | ± 9.50 |  | 175.54 | ± 6.91 | 357.48 | ± 8.77 |
| Goorsephid | 351.50 | ± 2.68 | 974.00 | | ± 22.00 |  | 239.35 | ± 1.46 | 661.34 | ± 3.13 |
| Hamashahr | 347.07 | ± 1.73 | 855.00 | | ± 5.00 |  | 309.66 | ± 0.97 | 673.61 | ± 7.88 |
| Hamedan | 365.66 | ± 4.47 | 828.00 | | ± 15.00 |  | 352.31 | ± 0.57 | 804.88 | ± 17.77 |
| HasanQeshlaq | 430.30 | ± 8.30 | 772.91 | | ± 0.50 |  | 314.86 | ± 12.23 | 558.50 | ± 0.36 |
| Heyran | 320.30 | ± 12.69 | 917.50 | | ± 14.50 |  | 239.53 | ± 10.85 | 546.73 | ± 13.21 |
| Hezarkanian | 448.40 | ± 6.60 | 776.11 | | ± 15.50 |  | 322.04 | ± 4.33 | 747.50 | ± 3.26 |
| Isparaxan | 323.47 | ± 1.06 | 988.00 | | ± 10.00 |  | 290.68 | ± 7.72 | 583.40 | ± 5.08 |
| Kalat | 343.83 | ± 3.83 | 791.50 | | ± 4.50 |  | 259.56 | ± 4.59 | 675.39 | ± 1.01 |
| KalatehNaqi | 321.57 | ± 1.54 | 784.50 | | ± 7.50 |  | 286.20 | ± 1.54 | 668.94 | ± 10.24 |
| KaniGanji | 326.99 | ± 6.04 | 844.00 | | ± 6.00 |  | 302.00 | ± 0.68 | 685.85 | ± 6.53 |
| Kargan | 351.60 | ± 0.23 | 934.50 | | ± 9.50 |  | 305.87 | ± 5.64 | 696.55 | ± 8.24 |
| Karimabad | 264.50 | ± 5.50 | 493.50 | | ± 7.50 |  | 200.34 | ± 1.3 | 445.92 | ± 3.97 |
| Karkaraq | 331.32 | ± 0.18 | 830.50 | | ± 4.50 |  | 266.39 | ± 0.49 | 680.01 | ± 6.33 |
| Karvandan | 505.58 | ± 2.62 | 1076.00 | | ± 5.00 |  | 381.24 | ± 2.97 | 820.46 | ± 4.20 |
| Kelardasht | 333.99 | ± 6.19 | 808.50 | | ± 4.50 |  | 269.04 | ± 8.07 | 645.10 | ± 7.60 |
| Khorramabad | 292.97 | ± 3.36 | 906.00 | | ± 2.00 |  | 257.10 | ± 5.00 | 587.46 | ± 5.84 |
| Khosrowabad | 351.75 | ± 1.25 | 842.00 | | ± 1.00 |  | 261.18 | ± 5.38 | 690.53 | ± 2.24 |
| KusehKahriz | 421.24 | ± 0.34 | 683.85 | | ± 8.00 |  | 289.96 | ± 18.23 | 570.00 | ± 0.90 |
| Laj | 333.56 | ± 7.55 | 810.00 | | ± 4.00 |  | 238.18 | ± 0.37 | 600.15 | ± 21.92 |
| Lamis | 438.50 | ± 3.50 | 944.50 | | ± 4.50 |  | 257.98 | ± 0.5 | 630.71 | ± 7.45 |
| LasemCheshmeh | 415.87 | ± 7.05 | 976.50 | | ± 0.50 |  | 291.15 | ± 7.94 | 669.90 | ± 15.25 |
| Liqvan | 292.12 | ± 4.42 | 1023.00 | | ± 16.00 |  | 237.56 | ± 5.84 | 565.99 | ± 5.63 |
| Losku | 260.73 | ± 2.44 | 470.00 | | ± 2.00 |  | 195.66 | ± 4.11 | 422.83 | ± 28.13 |
| Mamlejeh | 336.39 | ± 9.81 | 785.00 | | ± 2.00 |  | 256.82 | ± 3.11 | 668.79 | ± 0.03 |
| Marian | 278.55 | ± 3.08 | 622.00 | | ± 7.00 |  | 222.18 | ± 4.91 | 462.77 | ± 3.58 |
| MazraeBeed | 341.38 | ± 2.45 | 663.36 | | ± 11.13 |  | 209.55 | ± 10.12 | 559.88 | ± 0.61 |
| MihamlehyeOlya | 365.34 | ± 1.76 | 953.00 | | ± 1.00 |  | 258.87 | ± 2.17 | 656.68 | ± 12.04 |
| MirAzizi | 330.46 | ± 4.23 | 769.50 | | ± 5.50 |  | 300.76 | ± 6.33 | 652.57 | ± 5.81 |
| Naharkhoran | 358.71 | ± 8.78 | 662.26 | | ± 5.50 |  | 351.12 | ± 40.33 | 625.50 | ± 1.85 |
| Nasirabad | 367.58 | ± 7.54 | 1002.00 | | ± 9.00 |  | 223.05 | ± 2.96 | 628.01 | ± 20.68 |
| Noqan | 365.14 | ± 0.97 | 995.50 | | ± 5.50 |  | 224.88 | ± 1.56 | 536.50 | ± 7.40 |
| Nowgaran | 280.08 | ± 2.92 | 577.71 | | ± 9.00 |  | 258.64 | ± 0.96 | 461.00 | ± 1.44 |
| PahnehBar | 443.12 | ± 6.88 | 958.00 | | ± 2.00 |  | 274.63 | ± 1.54 | 494.26 | ± 4.00 |
| Palam | 316.85 | ± 6.76 | 786.00 | | ± 2.00 |  | 315.50 | ± 11.39 | 679.99 | ± 2.54 |
| Qorveh | 350.28 | ± 3.10 | 904.00 | | ± 0.00 |  | 313.55 | ± 5.36 | 652.86 | ± 8.62 |
| Qozivand | 350.12 | ± 5.80 | 853.00 | | ± 51.00 |  | 286.94 | ± 1.12 | 658.65 | ± 11.03 |
| QuriChay | 443.00 | ± 4.00 | 952.50 | | ± 2.50 |  | 308.82 | ± 5.02 | 430.41 | ± 1.27 |
| RezaqoliyeQeshlaq | 340.00 | ± 6.00 | 826.50 | | ± 3.50 |  | 269.36 | ± 2.68 | 697.64 | ± 9.30 |
| Roodafshan | 299.84 | ± 1.70 | 719.50 | | ± 0.50 |  | 244.38 | ± 2.31 | 640.59 | ± 3.48 |
| Sarab | 348.85 | ± 8.85 | 769.00 | | ± 0.00 |  | 304.36 | ± 4.34 | 540.22 | ± 16.51 |
| Sarbandan | 295.84 | ± 5.18 | 715.00 | | ± 10.00 |  | 229.12 | ± 1.73 | 535.57 | ± 16.95 |
| Seranza | 280.25 | ± 4.10 | 831.50 | | ± 9.50 |  | 227.74 | ± 3.70 | 534.16 | ± 18.32 |
| Shamasbi | 355.24 | ± 4.76 | 819.50 | | ± 1.50 |  | 263.54 | ± 7.70 | 677.45 | ± 2.06 |
| TangeSehRiz1 | 327.63 | ± 7.63 | 758.50 | | ± 2.50 |  | 315.02 | ± 0.82 | 721.01 | ± 11.06 |
| Sileh | 309.78 | ± 4.86 | 755.50 | | ± 9.50 |  | 268.00 | ± 10.76 | 606.14 | ± 15.16 |
| SinavaCheshme | 258.11 | ± 3.22 | 560.00 | | ± 4.00 |  | 205.36 | ± 4.36 | 448.65 | ± 4.55 |
| Subashi | 384.78 | ± 6.79 | 889.50 | | ± 15.50 |  | 246.66 | ± 1.73 | 530.14 | ± 9.37 |
| Sureshjan | 348.59 | ± 3.03 | 833.00 | | ± 4.00 |  | 250.73 | ± 7.10 | 538.03 | ± 17.02 |
| Talesh | 262.47 | ± 1.66 | 505.50 | | ± 4.50 |  | 194.56 | ± 3.87 | 492.68 | ± 12.6 |
| TangeSehRiz2 | 354.19 | ± 4.50 | 681.50 | | ± 6.50 |  | 321.79 | ± 7.35 | 483.53 | ± 11.69 |
| TangeTizab | 293.90 | ± 4.90 | 908.50 | | ± 3.50 |  | 238.67 | ± 0.29 | 528.30 | ± 2.46 |
| Tangrah | 381.22 | ± 6.22 | 885.50 | | ± 8.50 |  | 297.42 | ± 2.32 | 584.65 | ± 21.92 |
| Tazehabad | 246.25 | ± 3.90 | 526.00 | | ± 1.00 |  | 180.78 | ± 3.20 | 358.71 | ± 2.69 |
| Telochal | 304.04 | ± 4.00 | 893.50 | | ± 3.50 |  | 229.04 | ± 1.73 | 553.46 | ± 0.82 |
| Tokhmaqlu | 289.97 | ± 1.75 | 940.00 | | ± 9.00 |  | 231.06 | ± 4.97 | 643.62 | ± 11.67 |
| Torshab | 416.11 | ± 4.11 | 1005.50 | | ± 7.50 |  | 315.47 | ± 7.47 | 737.31 | ± 1.32 |
| Vanehbin | 333.68 | ± 8.07 | 919.50 | | ± 14.50 |  | 286.67 | ± 0.29 | 653.98 | ± 8.26 |
| Vila Darre Waterfall | 357.33 | ± 7.67 | 858.50 | | ± 8.50 |  | 336.68 | ± 2.73 | 750.71 | ± 4.74 |
| Yasuj | 382.83 | ± 4.63 | 607.78 | | ± 1.50 |  | 261.66 | ± 10.16 | 470.50 | ± 9.29 |
| Ziarat | 347.64 | ± 4.62 | 998.00 | | ± 12.00 |  | 218.97 | ± 2.76 | 542.42 | ± 1.92 |
| G | Non-stress | | | | |  | Drought stress | | | |
|  | 2018 | | 2019 | | |  | 2018 | | 2019 | |
|  | A | ± SE | A | | ± SE |  | A | ± SE | A | ± SE |
| Abbasabad | 11.34 | ± 0.16 | 12.56 | | ± 0.27 |  | 1.00 | ± 0.02 | 1.91 | ± 0.05 |
| Abr1 | 12.99 | ± 0.19 | 14.80 | | ± 0.30 |  | 1.10 | ± 0.10 | 1.69 | ± 0.05 |
| Abr1Forest1 | 13.29 | ± 0.09 | 15.29 | | ± 0.15 |  | 1.32 | ± 0.06 | 4.96 | ± 0.53 |
| Abr2 | 14.91 | ± 0.07 | 16.22 | | ± 0.05 |  | 3.90 | ± 0.06 | 6.53 | ± 0.50 |
| Abr2Forest2 | 12.47 | ± 0.33 | 15.58 | | ± 0.53 |  | 1.73 | ± 0.12 | 5.76 | ± 0.11 |
| Abr3Forest3 | 13.34 | ± 0.11 | 15.62 | | ± 0.47 |  | 3.02 | ± 0.10 | 4.23 | ± 0.11 |
| Abrumand | 14.74 | ± 0.04 | 17.25 | | ± 0.31 |  | 5.75 | ± 0.24 | 7.08 | ± 0.17 |
| Ahangaran | 13.37 | ± 0.36 | 15.80 | | ± 0.65 |  | 3.41 | ± 0.04 | 4.89 | ± 0.33 |
| Alvaresi | 12.25 | ± 0.09 | 17.13 | | ± 0.09 |  | 3.14 | ± 0.06 | 5.94 | ± 0.00 |
| Asadli | 14.45 | ± 0.12 | 16.20 | | ± 0.01 |  | 3.95 | ± 0.02 | 6.02 | ± 0.30 |
| Ashab | 11.05 | ± 0.03 | 15.46 | | ± 0.29 |  | 1.51 | ± 0.33 | 5.02 | ± 0.67 |
| Azizabad | 13.58 | ± 0.19 | 15.55 | | ± 0.51 |  | 2.62 | ± 0.26 | 5.39 | ± 0.06 |
| Aznãvleh | 13.41 | ± 0.06 | 15.31 | | ± 0.05 |  | 1.11 | ± 0.08 | 5.46 | ± 0.56 |
| BadKhoreh | 13.79 | ± 0.10 | 16.01 | | ± 0.15 |  | 3.47 | ± 0.22 | 2.03 | ± 0.02 |
| BandarehAnzali | 11.79 | ± 0.07 | 13.59 | | ± 0.76 |  | 1.17 | ± 0.11 | 1.52 | ± 0.04 |
| Baneh | 13.47 | ± 0.18 | 15.71 | | ± 0.03 |  | 3.11 | ± 0.01 | 1.70 | ± 0.03 |
| Basmenj | 14.62 | ± 0.36 | 16.99 | | ± 0.03 |  | 4.09 | ± 0.07 | 6.62 | ± 0.00 |
| Beyraq | 12.43 | ± 0.14 | 13.96 | | ± 0.05 |  | 1.59 | ± 0.25 | 5.57 | ± 0.07 |
| Bisotun | 13.19 | ± 0.15 | 15.92 | | ± 0.03 |  | 2.85 | ± 0.01 | 5.23 | ± 0.34 |
| Borbor | 13.02 | ± 0.12 | 15.00 | | ± 0.03 |  | 2.74 | ± 0.07 | 2.19 | ± 0.16 |
| Borhan | 14.08 | ± 0.30 | 16.01 | | ± 0.03 |  | 4.04 | ± 0.65 | 6.07 | ± 0.35 |
| Chadegan | 13.51 | ± 0.04 | 15.45 | | ± 0.19 |  | 3.46 | ± 0.17 | 5.10 | ± 0.57 |
| Chaleki | 11.62 | ± 0.18 | 13.47 | | ± 0.10 |  | 1.19 | ± 0.05 | 1.77 | ± 0.02 |
| Chali | 13.20 | ± 0.23 | 14.79 | | ± 0.06 |  | 2.72 | ± 0.19 | 4.80 | ± 0.44 |
| Chavarchin | 13.65 | ± 0.11 | 15.67 | | ± 0.39 |  | 3.09 | ± 0.09 | 4.89 | ± 0.38 |
| Ciakhor | 14.76 | ± 0.04 | 18.22 | | ± 0.24 |  | 4.29 | ± 0.27 | 6.87 | ± 0.03 |
| Damavand | 14.25 | ± 0.53 | 16.91 | | ± 0.07 |  | 4.93 | ± 0.09 | 6.95 | ± 0.04 |
| Damghanat | 13.60 | ± 0.05 | 14.84 | | ± 0.18 |  | 3.00 | ± 0.09 | 4.78 | ± 0.01 |
| Darband | 13.56 | ± 0.06 | 14.54 | | ± 0.51 |  | 3.11 | ± 0.04 | 4.02 | ± 0.00 |
| DareSari | 12.27 | ± 0.07 | 16.89 | | ± 0.65 |  | 1.19 | ± 0.16 | 3.86 | ± 0.39 |
| Darman | 13.80 | ± 0.23 | 15.29 | | ± 0.08 |  | 3.26 | ± 0.11 | 1.64 | ± 0.13 |
| DarrehLak | 13.71 | ± 0.06 | 15.72 | | ± 0.03 |  | 4.20 | ± 0.23 | 5.13 | ± 0.29 |
| DoSar | 13.82 | ± 0.13 | 16.61 | | ± 0.16 |  | 4.20 | ± 0.44 | 6.84 | ± 0.00 |
| Dowlatabad | 13.70 | ± 0.07 | 14.92 | | ± 0.04 |  | 1.86 | ± 0.22 | 5.91 | ± 0.07 |
| Duzduzan | 13.45 | ± 0.12 | 16.14 | | ± 0.10 |  | 3.21 | ± 0.30 | 6.06 | ± 0.02 |
| EisaKand | 13.78 | ± 0.21 | 14.53 | | ± 0.07 |  | 3.35 | ± 0.33 | 6.07 | ± 0.08 |
| Filabad | 12.12 | ± 0.02 | 13.79 | | ± 0.25 |  | 2.51 | ± 0.12 | 3.80 | ± 0.15 |
| Gaznaq | 13.51 | ± 0.09 | 16.13 | | ± 0.19 |  | 3.89 | ± 0.15 | 5.95 | ± 0.07 |
| Ghircanyon1 | 11.63 | ± 0.41 | 14.21 | | ± 0.28 |  | 1.42 | ± 0.05 | 3.29 | ± 0.93 |
| GilanTappeh | 11.83 | ± 0.42 | 13.61 | | ± 0.14 |  | 0.96 | ± 0.03 | 1.52 | ± 0.23 |
| Goorsephid | 12.21 | ± 0.08 | 16.79 | | ± 0.25 |  | 1.35 | ± 0.03 | 5.61 | ± 0.07 |
| Hamashahr | 13.57 | ± 0.15 | 14.12 | | ± 0.09 |  | 3.48 | ± 0.14 | 5.65 | ± 0.31 |
| Hamedan | 13.67 | ± 0.35 | 15.94 | | ± 0.04 |  | 4.51 | ± 0.24 | 6.62 | ± 0.03 |
| HasanQeshlaq | 13.34 | ± 0.15 | 16.56 | | ± 0.32 |  | 3.96 | ± 0.02 | 6.23 | ± 0.02 |
| Heyran | 12.79 | ± 0.11 | 14.48 | | ± 0.11 |  | 1.42 | ± 0.15 | 3.43 | ± 0.76 |
| Hezarkanian | 14.66 | ± 0.23 | 16.57 | | ± 0.31 |  | 4.40 | ± 0.17 | 6.35 | ± 0.00 |
| Isparaxan | 12.18 | ± 0.04 | 16.58 | | ± 0.18 |  | 3.02 | ± 0.09 | 4.52 | ± 0.48 |
| Kalat | 13.33 | ± 0.45 | 14.18 | | ± 0.17 |  | 3.34 | ± 0.33 | 4.82 | ± 0.14 |
| KalatehNaqi | 12.27 | ± 0.12 | 14.65 | | ± 0.10 |  | 3.30 | ± 0.09 | 5.12 | ± 0.14 |
| KaniGanji | 13.06 | ± 0.02 | 15.13 | | ± 0.11 |  | 2.82 | ± 0.19 | 3.82 | ± 0.13 |
| Kargan | 13.15 | ± 0.15 | 16.41 | | ± 0.38 |  | 3.73 | ± 0.14 | 5.29 | ± 0.41 |
| Karimabad | 12.03 | ± 0.01 | 13.65 | | ± 0.22 |  | 1.16 | ± 0.03 | 1.71 | ± 0.10 |
| Karkaraq | 12.95 | ± 0.57 | 16.35 | | ± 0.92 |  | 2.02 | ± 0.97 | 4.78 | ± 0.14 |
| Karvandan | 14.24 | ± 0.22 | 17.13 | | ± 0.19 |  | 3.26 | ± 1.21 | 6.70 | ± 0.02 |
| Kelardasht | 13.65 | ± 0.07 | 14.14 | | ± 0.12 |  | 3.42 | ± 0.85 | 4.81 | ± 0.07 |
| Khorramabad | 13.30 | ± 0.80 | 14.67 | | ± 0.19 |  | 3.04 | ± 0.11 | 4.30 | ± 0.22 |
| Khosrowabad | 12.91 | ± 0.52 | 15.13 | | ± 0.83 |  | 2.66 | ± 1.36 | 5.22 | ± 0.17 |
| KusehKahriz | 12.27 | ± 0.12 | 13.93 | | ± 0.59 |  | 2.91 | ± 0.06 | 5.18 | ± 0.35 |
| Laj | 12.76 | ± 0.26 | 14.89 | | ± 0.41 |  | 2.56 | ± 0.45 | 4.39 | ± 0.24 |
| Lamis | 12.30 | ± 0.04 | 14.15 | | ± 0.07 |  | 3.05 | ± 0.02 | 4.84 | ± 0.21 |
| LasemCheshmeh | 14.41 | ± 0.27 | 14.88 | | ± 0.29 |  | 3.37 | ± 0.24 | 6.50 | ± 0.35 |
| Liqvan | 12.22 | ± 0.24 | 17.42 | | ± 0.10 |  | 2.37 | ± 0.95 | 3.12 | ± 1.10 |
| Losku | 11.36 | ± 0.16 | 13.12 | | ± 0.15 |  | 1.16 | ± 0.02 | 1.68 | ± 0.27 |
| Mamlejeh | 13.58 | ± 0.11 | 15.95 | | ± 0.26 |  | 3.01 | ± 0.03 | 2.67 | ± 0.36 |
| Marian | 11.97 | ± 0.41 | 14.40 | | ± 0.39 |  | 1.09 | ± 0.03 | 2.05 | ± 0.02 |
| MazraeBeed | 11.15 | ± 0.02 | 13.40 | | ± 0.04 |  | 2.81 | ± 0.76 | 4.11 | ± 0.15 |
| MihamlehyeOlya | 13.38 | ± 0.40 | 17.12 | | ± 0.10 |  | 3.31 | ± 0.11 | 4.17 | ± 0.05 |
| MirAzizi | 13.33 | ± 0.24 | 14.67 | | ± 0.39 |  | 2.85 | ± 0.10 | 5.11 | ± 0.27 |
| Naharkhoran | 12.16 | ± 0.69 | 13.46 | | ± 0.45 |  | 3.91 | ± 0.32 | 3.32 | ± 0.02 |
| Nasirabad | 12.94 | ± 0.47 | 16.95 | | ± 0.38 |  | 1.28 | ± 0.06 | 5.23 | ± 0.32 |
| Noqan | 12.30 | ± 0.17 | 17.25 | | ± 0.01 |  | 1.58 | ± 0.32 | 2.21 | ± 0.15 |
| Nowgaran | 13.33 | ± 0.11 | 13.29 | | ± 0.05 |  | 3.72 | ± 0.04 | 3.81 | ± 0.32 |
| PahnehBar | 13.30 | ± 0.51 | 15.15 | | ± 0.16 |  | 3.00 | ± 0.02 | 1.52 | ± 0.05 |
| Palam | 13.12 | ± 0.13 | 16.05 | | ± 0.15 |  | 2.74 | ± 0.00 | 5.21 | ± 0.14 |
| Qorveh | 13.00 | ± 0.02 | 16.42 | | ± 0.38 |  | 4.21 | ± 0.06 | 3.68 | ± 0.33 |
| Qozivand | 13.75 | ± 0.22 | 15.47 | | ± 0.94 |  | 3.03 | ± 0.13 | 4.81 | ± 0.45 |
| QuriChay | 13.48 | ± 0.51 | 16.15 | | ± 0.87 |  | 3.92 | ± 0.34 | 1.57 | ± 0.02 |
| RezaqoliyeQeshlaq | 13.47 | ± 0.41 | 16.02 | | ± 0.35 |  | 3.86 | ± 0.28 | 5.27 | ± 0.32 |
| Roodafshan | 13.53 | ± 0.08 | 13.76 | | ± 0.34 |  | 2.99 | ± 0.07 | 4.92 | ± 0.34 |
| Sarab | 13.79 | ± 0.20 | 15.89 | | ± 0.18 |  | 3.69 | ± 0.09 | 2.01 | ± 0.03 |
| Sarbandan | 12.66 | ± 0.60 | 15.34 | | ± 0.43 |  | 3.08 | ± 0.29 | 5.57 | ± 0.06 |
| Seranza | 12.00 | ± 0.02 | 14.42 | | ± 0.21 |  | 1.41 | ± 0.37 | 2.43 | ± 0.31 |
| Shamasbi | 13.05 | ± 0.03 | 15.54 | | ± 0.22 |  | 3.89 | ± 0.18 | 5.19 | ± 0.17 |
| TangeSehRiz1 | 12.89 | ± 0.10 | 13.76 | | ± 0.25 |  | 3.44 | ± 0.07 | 6.26 | ± 0.09 |
| Sileh | 13.37 | ± 0.07 | 14.02 | | ± 0.11 |  | 2.51 | ± 0.39 | 4.85 | ± 0.18 |
| SinavaCheshme | 11.36 | ± 0.21 | 13.75 | | ± 0.14 |  | 1.12 | ± 0.03 | 5.86 | ± 0.06 |
| Subashi | 12.25 | ± 0.17 | 16.78 | | ± 0.24 |  | 2.27 | ± 0.76 | 3.15 | ± 0.70 |
| Sureshjan | 13.42 | ± 0.17 | 14.77 | | ± 0.25 |  | 2.90 | ± 0.14 | 3.16 | ± 0.79 |
| Talesh | 11.64 | ± 0.13 | 13.46 | | ± 0.04 |  | 1.25 | ± 0.00 | 1.56 | ± 0.21 |
| TangeSehRiz2 | 13.77 | ± 0.20 | 16.01 | | ± 0.03 |  | 3.94 | ± 0.15 | 1.84 | ± 0.00 |
| TangeTizab | 13.25 | ± 0.02 | 15.80 | | ± 0.03 |  | 2.13 | ± 0.64 | 5.29 | ± 0.14 |
| Tangrah | 12.22 | ± 0.04 | 13.79 | | ± 0.34 |  | 3.02 | ± 0.24 | 3.92 | ± 0.23 |
| Tazehabad | 11.77 | ± 0.35 | 13.47 | | ± 0.21 |  | 1.05 | ± 0.03 | 1.64 | ± 0.28 |
| Telochal | 13.16 | ± 0.19 | 15.76 | | ± 0.24 |  | 1.80 | ± 0.73 | 2.50 | ± 0.47 |
| Tokhmaqlu | 11.97 | ± 0.09 | 14.01 | | ± 0.01 |  | 3.10 | ± 0.13 | 4.92 | ± 0.41 |
| Torshab | 13.88 | ± 0.68 | 16.15 | | ± 0.21 |  | 3.93 | ± 0.19 | 5.91 | ± 0.07 |
| Vanehbin | 12.06 | ± 0.23 | 14.31 | | ± 0.08 |  | 3.39 | ± 0.02 | 5.02 | ± 0.02 |
| Vila Darre Waterfall | 13.50 | ± 0.05 | 16.50 | | ± 0.21 |  | 3.88 | ± 0.07 | 6.30 | ± 0.25 |
| Yasuj | 14.11 | ± 0.43 | 13.68 | | ± 0.05 |  | 2.49 | ± 0.52 | 5.40 | ± 0.24 |
| Ziarat | 12.51 | ± 0.07 | 13.75 | | ± 0.70 |  | 1.19 | ± 0.04 | 3.90 | ± 0.06 |
| G | Non-stress | | | | |  | Drought stress | | | |
|  | 2018 | | 2019 | | |  | 2018 | | 2019 | |
|  | gs | ± SE | gs | | ± SE |  | gs | ± SE | gs | ± SE |
| Abbasabad | 0.18 | ± 0.0035 | 0.20 | | ± 0.0015 |  | 0.01 | ± 0.0005 | 0.03 | ± 0.0050 |
| Abr1 | 0.22 | ± 0.0090 | 0.25 | | ± 0.0090 |  | 0.01 | ± 0.0017 | 0.03 | ± 0.0050 |
| Abr1Forest1 | 0.21 | ± 0.0130 | 0.24 | | ± 0.0115 |  | 0.06 | ± 0.0001 | 0.07 | ± 0.0031 |
| Abr2 | 0.32 | ± 0.0050 | 0.32 | | ± 0.0250 |  | 0.12 | ± 0.0040 | 0.11 | ± 0.0285 |
| Abr2Forest2 | 0.21 | ± 0.0020 | 0.26 | | ± 0.0075 |  | 0.03 | ± 0.0003 | 0.06 | ± 0.0009 |
| Abr3Forest3 | 0.21 | ± 0.0135 | 0.26 | | ± 0.0285 |  | 0.05 | ± 0.0011 | 0.05 | ± 0.0004 |
| Abrumand | 0.31 | ± 0.0035 | 0.35 | | ± 0.0085 |  | 0.12 | ± 0.0005 | 0.15 | ± 0.0408 |
| Ahangaran | 0.22 | ± 0.0175 | 0.25 | | ± 0.0065 |  | 0.05 | ± 0.0001 | 0.06 | ± 0.0004 |
| Alvaresi | 0.22 | ± 0.0035 | 0.35 | | ± 0.0100 |  | 0.03 | ± 0.0017 | 0.05 | ± 0.0001 |
| Asadli | 0.21 | ± 0.0035 | 0.25 | | ± 0.0035 |  | 0.08 | ± 0.0009 | 0.08 | ± 0.0074 |
| Ashab | 0.17 | ± 0.0030 | 0.24 | | ± 0.0020 |  | 0.03 | ± 0.0069 | 0.06 | ± 0.0030 |
| Azizabad | 0.21 | ± 0.0005 | 0.25 | | ± 0.0105 |  | 0.06 | ± 0.0007 | 0.08 | ± 0.0020 |
| Aznãvleh | 0.22 | ± 0.0005 | 0.25 | | ± 0.0125 |  | 0.02 | ± 0.0050 | 0.08 | ± 0.0020 |
| BadKhoreh | 0.27 | ± 0.0070 | 0.29 | | ± 0.0075 |  | 0.06 | ± 0.0008 | 0.04 | ± 0.0005 |
| BandarehAnzali | 0.20 | ± 0.0205 | 0.23 | | ± 0.0013 |  | 0.01 | ± 0.0001 | 0.04 | ± 0.0005 |
| Baneh | 0.22 | ± 0.0047 | 0.26 | | ± 0.0065 |  | 0.07 | ± 0.0000 | 0.09 | ± 0.0013 |
| Basmenj | 0.31 | ± 0.0000 | 0.33 | | ± 0.0160 |  | 0.07 | ± 0.0044 | 0.09 | ± 0.0056 |
| Beyraq | 0.21 | ± 0.0015 | 0.24 | | ± 0.0045 |  | 0.03 | ± 0.0010 | 0.05 | ± 0.0000 |
| Bisotun | 0.21 | ± 0.0150 | 0.25 | | ± 0.0090 |  | 0.05 | ± 0.0099 | 0.07 | ± 0.0020 |
| Borbor | 0.22 | ± 0.0020 | 0.26 | | ± 0.0160 |  | 0.06 | ± 0.0038 | 0.08 | ± 0.0012 |
| Borhan | 0.30 | ± 0.0010 | 0.31 | | ± 0.0035 |  | 0.03 | ± 0.0007 | 0.11 | ± 0.0070 |
| Chadegan | 0.21 | ± 0.0145 | 0.24 | | ± 0.0120 |  | 0.04 | ± 0.0024 | 0.05 | ± 0.0009 |
| Chaleki | 0.19 | ± 0.0000 | 0.22 | | ± 0.0020 |  | 0.01 | ± 0.0019 | 0.04 | ± 0.0015 |
| Chali | 0.22 | ± 0.0105 | 0.26 | | ± 0.0125 |  | 0.05 | ± 0.0070 | 0.07 | ± 0.0066 |
| Chavarchin | 0.22 | ± 0.0100 | 0.27 | | ± 0.0105 |  | 0.06 | ± 0.0046 | 0.08 | ± 0.0014 |
| Ciakhor | 0.31 | ± 0.0005 | 0.38 | | ± 0.0030 |  | 0.10 | ± 0.0130 | 0.13 | ± 0.0000 |
| Damavand | 0.29 | ± 0.0070 | 0.33 | | ± 0.0035 |  | 0.10 | ± 0.0005 | 0.12 | ± 0.0110 |
| Damghanat | 0.22 | ± 0.0025 | 0.26 | | ± 0.0005 |  | 0.05 | ± 0.0067 | 0.07 | ± 0.0009 |
| Darband | 0.21 | ± 0.0105 | 0.24 | | ± 0.0050 |  | 0.06 | ± 0.0044 | 0.05 | ± 0.0009 |
| DareSari | 0.22 | ± 0.0200 | 0.34 | | ± 0.0050 |  | 0.06 | ± 0.0052 | 0.05 | ± 0.0042 |
| Darman | 0.29 | ± 0.0030 | 0.34 | | ± 0.0035 |  | 0.04 | ± 0.0002 | 0.03 | ± 0.0055 |
| DarrehLak | 0.20 | ± 0.0065 | 0.24 | | ± 0.0045 |  | 0.08 | ± 0.0023 | 0.08 | ± 0.0007 |
| DoSar | 0.30 | ± 0.0010 | 0.31 | | ± 0.0045 |  | 0.10 | ± 0.0015 | 0.11 | ± 0.0170 |
| Dowlatabad | 0.21 | ± 0.0065 | 0.25 | | ± 0.0090 |  | 0.06 | ± 0.0026 | 0.08 | ± 0.0012 |
| Duzduzan | 0.19 | ± 0.0045 | 0.25 | | ± 0.0185 |  | 0.07 | ± 0.0038 | 0.10 | ± 0.0203 |
| EisaKand | 0.21 | ± 0.0135 | 0.24 | | ± 0.0055 |  | 0.06 | ± 0.0006 | 0.08 | ± 0.0001 |
| Filabad | 0.21 | ± 0.0100 | 0.26 | | ± 0.0080 |  | 0.03 | ± 0.0018 | 0.05 | ± 0.0025 |
| Gaznaq | 0.20 | ± 0.0065 | 0.26 | | ± 0.0230 |  | 0.06 | ± 0.0006 | 0.08 | ± 0.0004 |
| Ghircanyon1 | 0.19 | ± 0.0125 | 0.23 | | ± 0.0050 |  | 0.06 | ± 0.0038 | 0.04 | ± 0.0030 |
| GilanTappeh | 0.22 | ± 0.0200 | 0.25 | | ± 0.0130 |  | 0.02 | ± 0.0009 | 0.02 | ± 0.0006 |
| Goorsephid | 0.27 | ± 0.0040 | 0.32 | | ± 0.0065 |  | 0.04 | ± 0.0093 | 0.06 | ± 0.0045 |
| Hamashahr | 0.22 | ± 0.0005 | 0.25 | | ± 0.0110 |  | 0.06 | ± 0.0053 | 0.09 | ± 0.0008 |
| Hamedan | 0.21 | ± 0.0050 | 0.25 | | ± 0.0055 |  | 0.11 | ± 0.0026 | 0.12 | ± 0.0030 |
| HasanQeshlaq | 0.20 | ± 0.0040 | 0.23 | | ± 0.0075 |  | 0.07 | ± 0.0010 | 0.09 | ± 0.0012 |
| Heyran | 0.20 | ± 0.0000 | 0.24 | | ± 0.0015 |  | 0.03 | ± 0.0019 | 0.04 | ± 0.0001 |
| Hezarkanian | 0.31 | ± 0.0035 | 0.35 | | ± 0.0050 |  | 0.08 | ± 0.0006 | 0.10 | ± 0.0070 |
| Isparaxan | 0.28 | ± 0.0060 | 0.32 | | ± 0.0054 |  | 0.06 | ± 0.0019 | 0.07 | ± 0.0032 |
| Kalat | 0.20 | ± 0.0065 | 0.23 | | ± 0.0005 |  | 0.05 | ± 0.0037 | 0.07 | ± 0.0012 |
| KalatehNaqi | 0.21 | ± 0.0170 | 0.26 | | ± 0.0150 |  | 0.04 | ± 0.0095 | 0.07 | ± 0.0026 |
| KaniGanji | 0.21 | ± 0.0055 | 0.25 | | ± 0.0010 |  | 0.06 | ± 0.0031 | 0.08 | ± 0.0002 |
| Kargan | 0.20 | ± 0.0060 | 0.24 | | ± 0.0075 |  | 0.04 | ± 0.0019 | 0.08 | ± 0.0008 |
| Karimabad | 0.20 | ± 0.0075 | 0.24 | | ± 0.0010 |  | 0.01 | ± 0.0004 | 0.03 | ± 0.0040 |
| Karkaraq | 0.20 | ± 0.0070 | 0.25 | | ± 0.0165 |  | 0.05 | ± 0.0019 | 0.07 | ± 0.0009 |
| Karvandan | 0.29 | ± 0.0045 | 0.35 | | ± 0.0015 |  | 0.09 | ± 0.0031 | 0.14 | ± 0.0010 |
| Kelardasht | 0.21 | ± 0.0090 | 0.24 | | ± 0.0040 |  | 0.04 | ± 0.0034 | 0.06 | ± 0.0001 |
| Khorramabad | 0.19 | ± 0.0090 | 0.24 | | ± 0.0030 |  | 0.07 | ± 0.0055 | 0.06 | ± 0.0040 |
| Khosrowabad | 0.22 | ± 0.0040 | 0.26 | | ± 0.0075 |  | 0.04 | ± 0.0066 | 0.08 | ± 0.0017 |
| KusehKahriz | 0.21 | ± 0.0010 | 0.23 | | ± 0.0050 |  | 0.04 | ± 0.0056 | 0.07 | ± 0.0008 |
| Laj | 0.20 | ± 0.0085 | 0.25 | | ± 0.0055 |  | 0.06 | ± 0.0020 | 0.05 | ± 0.0017 |
| Lamis | 0.20 | ± 0.0070 | 0.25 | | ± 0.0040 |  | 0.05 | ± 0.0090 | 0.06 | ± 0.0118 |
| LasemCheshmeh | 0.30 | ± 0.0020 | 0.32 | | ± 0.0005 |  | 0.07 | ± 0.0013 | 0.09 | ± 0.0050 |
| Liqvan | 0.28 | ± 0.0055 | 0.37 | | ± 0.0020 |  | 0.03 | ± 0.0031 | 0.04 | ± 0.0005 |
| Losku | 0.19 | ± 0.0045 | 0.21 | | ± 0.0025 |  | 0.02 | ± 0.0027 | 0.02 | ± 0.0003 |
| Mamlejeh | 0.20 | ± 0.0050 | 0.24 | | ± 0.0065 |  | 0.04 | ± 0.0105 | 0.06 | ± 0.0042 |
| Marian | 0.19 | ± 0.0010 | 0.24 | | ± 0.0035 |  | 0.02 | ± 0.0023 | 0.04 | ± 0.0004 |
| MazraeBeed | 0.17 | ± 0.0040 | 0.22 | | ± 0.0015 |  | 0.03 | ± 0.0017 | 0.05 | ± 0.0037 |
| MihamlehyeOlya | 0.26 | ± 0.0075 | 0.34 | | ± 0.0050 |  | 0.05 | ± 0.0059 | 0.05 | ± 0.0001 |
| MirAzizi | 0.20 | ± 0.0030 | 0.24 | | ± 0.0070 |  | 0.05 | ± 0.0014 | 0.07 | ± 0.0037 |
| Naharkhoran | 0.20 | ± 0.0045 | 0.24 | | ± 0.0000 |  | 0.04 | ± 0.0055 | 0.08 | ± 0.0087 |
| Nasirabad | 0.20 | ± 0.0020 | 0.35 | | ± 0.0020 |  | 0.03 | ± 0.0104 | 0.08 | ± 0.0037 |
| Noqan | 0.24 | ± 0.0055 | 0.36 | | ± 0.0065 |  | 0.03 | ± 0.0011 | 0.04 | ± 0.0030 |
| Nowgaran | 0.20 | ± 0.0040 | 0.22 | | ± 0.0010 |  | 0.04 | ± 0.0039 | 0.06 | ± 0.0083 |
| PahnehBar | 0.20 | ± 0.0010 | 0.24 | | ± 0.0015 |  | 0.06 | ± 0.0025 | 0.03 | ± 0.0020 |
| Palam | 0.20 | ± 0.0005 | 0.26 | | ± 0.0200 |  | 0.06 | ± 0.0016 | 0.07 | ± 0.0035 |
| Qorveh | 0.22 | ± 0.0145 | 0.25 | | ± 0.0020 |  | 0.06 | ± 0.0010 | 0.08 | ± 0.0012 |
| Qozivand | 0.22 | ± 0.0125 | 0.26 | | ± 0.0135 |  | 0.05 | ± 0.0023 | 0.06 | ± 0.0012 |
| QuriChay | 0.21 | ± 0.0040 | 0.25 | | ± 0.0105 |  | 0.07 | ± 0.0047 | 0.03 | ± 0.0025 |
| RezaqoliyeQeshlaq | 0.21 | ± 0.0075 | 0.24 | | ± 0.0080 |  | 0.06 | ± 0.0012 | 0.08 | ± 0.0021 |
| Roodafshan | 0.20 | ± 0.0055 | 0.24 | | ± 0.0065 |  | 0.04 | ± 0.0067 | 0.06 | ± 0.0038 |
| Sarab | 0.27 | ± 0.0075 | 0.29 | | ± 0.0020 |  | 0.06 | ± 0.0031 | 0.04 | ± 0.0055 |
| Sarbandan | 0.20 | ± 0.0125 | 0.27 | | ± 0.0115 |  | 0.03 | ± 0.0003 | 0.04 | ± 0.0000 |
| Seranza | 0.20 | ± 0.0025 | 0.25 | | ± 0.0090 |  | 0.03 | ± 0.0021 | 0.04 | ± 0.0025 |
| Shamasbi | 0.23 | ± 0.0080 | 0.25 | | ± 0.0165 |  | 0.05 | ± 0.0031 | 0.07 | ± 0.0069 |
| TangeSehRiz1 | 0.22 | ± 0.0100 | 0.25 | | ± 0.0020 |  | 0.06 | ± 0.0037 | 0.09 | ± 0.0038 |
| Sileh | 0.21 | ± 0.0150 | 0.26 | | ± 0.0040 |  | 0.04 | ± 0.0033 | 0.06 | ± 0.0035 |
| SinavaCheshme | 0.18 | ± 0.0010 | 0.24 | | ± 0.0050 |  | 0.01 | ± 0.0017 | 0.05 | ± 0.0001 |
| Subashi | 0.26 | ± 0.0070 | 0.32 | | ± 0.0060 |  | 0.04 | ± 0.0011 | 0.04 | ± 0.0008 |
| Sureshjan | 0.22 | ± 0.0030 | 0.26 | | ± 0.0075 |  | 0.04 | ± 0.0005 | 0.05 | ± 0.0048 |
| Talesh | 0.20 | ± 0.0060 | 0.22 | | ± 0.0025 |  | 0.01 | ± 0.0039 | 0.03 | ± 0.0040 |
| TangeSehRiz2 | 0.21 | ± 0.0035 | 0.25 | | ± 0.0050 |  | 0.07 | ± 0.0025 | 0.04 | ± 0.0010 |
| TangeTizab | 0.20 | ± 0.0015 | 0.29 | | ± 0.0040 |  | 0.03 | ± 0.0030 | 0.04 | ± 0.0020 |
| Tangrah | 0.23 | ± 0.0150 | 0.25 | | ± 0.0000 |  | 0.05 | ± 0.0048 | 0.06 | ± 0.0048 |
| Tazehabad | 0.18 | ± 0.0025 | 0.22 | | ± 0.0015 |  | 0.01 | ± 0.0001 | 0.02 | ± 0.0005 |
| Telochal | 0.20 | ± 0.0075 | 0.24 | | ± 0.0000 |  | 0.04 | ± 0.0064 | 0.05 | ± 0.0022 |
| Tokhmaqlu | 0.21 | ± 0.0075 | 0.24 | | ± 0.0035 |  | 0.06 | ± 0.0051 | 0.07 | ± 0.0001 |
| Torshab | 0.28 | ± 0.0060 | 0.28 | | ± 0.0110 |  | 0.07 | ± 0.0047 | 0.09 | ± 0.0067 |
| Vanehbin | 0.20 | ± 0.0060 | 0.26 | | ± 0.0105 |  | 0.04 | ± 0.0034 | 0.06 | ± 0.0027 |
| Vila Darre Waterfall | 0.22 | ± 0.0100 | 0.24 | | ± 0.0030 |  | 0.07 | ± 0.0001 | 0.10 | ± 0.0105 |
| Yasuj | 0.25 | ± 0.0340 | 0.23 | | ± 0.0035 |  | 0.03 | ± 0.0027 | 0.06 | ± 0.0034 |
| Ziarat | 0.20 | ± 0.0060 | 0.24 | | ± 0.0095 |  | 0.02 | ± 0.0034 | 0.03 | ± 0.0005 |
| G | Non-stress | | | | |  | Drought stress | | | |
|  | 2018 | | 2019 | | |  | 2018 | | 2019 | |
|  | Tr | ± SE | Tr | | ± SE |  | Tr | ± SE | Tr | ± SE |
| Abbasabad | 2.92 | ± 0.046 | 2.98 | | ± 0.075 |  | 0.35 | ± 0.151 | 0.55 | ± 0.015 |
| Abr1 | 3.33 | ± 0.038 | 3.79 | | ± 0.084 |  | 0.50 | ± 0.088 | 0.79 | ± 0.189 |
| Abr1Forest1 | 3.47 | ± 0.020 | 3.84 | | ± 0.006 |  | 0.83 | ± 0.002 | 1.00 | ± 0.013 |
| Abr2 | 4.16 | ± 0.037 | 4.98 | | ± 0.042 |  | 1.47 | ± 0.070 | 1.64 | ± 0.315 |
| Abr2Forest2 | 3.28 | ± 0.112 | 3.69 | | ± 0.042 |  | 0.70 | ± 0.016 | 1.09 | ± 0.044 |
| Abr3Forest3 | 3.36 | ± 0.041 | 3.72 | | ± 0.002 |  | 0.78 | ± 0.043 | 1.00 | ± 0.012 |
| Abrumand | 4.17 | ± 0.001 | 4.45 | | ± 0.044 |  | 1.51 | ± 0.091 | 0.97 | ± 0.550 |
| Ahangaran | 3.70 | ± 0.043 | 3.82 | | ± 0.072 |  | 0.76 | ± 0.003 | 1.01 | ± 0.001 |
| Alvaresi | 3.73 | ± 0.006 | 4.31 | | ± 0.055 |  | 0.69 | ± 0.027 | 0.92 | ± 0.003 |
| Asadli | 3.72 | ± 0.035 | 3.86 | | ± 0.013 |  | 1.25 | ± 0.118 | 1.17 | ± 0.130 |
| Ashab | 2.72 | ± 0.075 | 3.37 | | ± 0.045 |  | 0.65 | ± 0.007 | 1.03 | ± 0.036 |
| Azizabad | 3.73 | ± 0.040 | 3.96 | | ± 0.025 |  | 0.90 | ± 0.015 | 1.38 | ± 0.094 |
| Aznãvleh | 3.49 | ± 0.004 | 3.72 | | ± 0.023 |  | 0.62 | ± 0.001 | 0.98 | ± 0.001 |
| BadKhoreh | 3.40 | ± 0.006 | 3.92 | | ± 0.003 |  | 0.83 | ± 0.069 | 1.35 | ± 0.036 |
| BandarehAnzali | 2.98 | ± 0.002 | 3.18 | | ± 0.096 |  | 0.64 | ± 0.004 | 0.91 | ± 0.005 |
| Baneh | 3.47 | ± 0.028 | 3.92 | | ± 0.035 |  | 1.23 | ± 0.101 | 0.78 | ± 0.190 |
| Basmenj | 4.09 | ± 0.006 | 4.43 | | ± 0.010 |  | 1.12 | ± 0.145 | 1.60 | ± 0.018 |
| Beyraq | 3.83 | ± 0.050 | 3.98 | | ± 0.033 |  | 0.67 | ± 0.007 | 1.54 | ± 0.012 |
| Bisotun | 3.62 | ± 0.019 | 3.99 | | ± 0.036 |  | 0.82 | ± 0.019 | 0.97 | ± 0.002 |
| Borbor | 3.67 | ± 0.041 | 3.96 | | ± 0.060 |  | 0.83 | ± 0.033 | 1.31 | ± 0.018 |
| Borhan | 3.99 | ± 0.007 | 4.19 | | ± 0.050 |  | 0.95 | ± 0.022 | 1.61 | ± 0.077 |
| Chadegan | 3.75 | ± 0.004 | 4.00 | | ± 0.014 |  | 0.74 | ± 0.002 | 1.10 | ± 0.029 |
| Chaleki | 2.91 | ± 0.016 | 3.04 | | ± 0.001 |  | 0.61 | ± 0.007 | 0.88 | ± 0.010 |
| Chali | 3.35 | ± 0.049 | 3.92 | | ± 0.066 |  | 0.79 | ± 0.031 | 1.19 | ± 0.095 |
| Chavarchin | 3.69 | ± 0.012 | 3.90 | | ± 0.113 |  | 0.85 | ± 0.062 | 0.96 | ± 0.000 |
| Ciakhor | 4.16 | ± 0.006 | 4.52 | | ± 0.034 |  | 1.45 | ± 0.010 | 1.72 | ± 0.020 |
| Damavand | 4.17 | ± 0.007 | 4.33 | | ± 0.036 |  | 1.54 | ± 0.043 | 1.82 | ± 0.028 |
| Damghanat | 3.76 | ± 0.103 | 4.01 | | ± 0.025 |  | 0.83 | ± 0.020 | 1.10 | ± 0.007 |
| Darband | 3.38 | ± 0.004 | 3.73 | | ± 0.043 |  | 1.01 | ± 0.124 | 0.98 | ± 0.010 |
| DareSari | 3.88 | ± 0.056 | 4.22 | | ± 0.010 |  | 0.69 | ± 0.013 | 1.17 | ± 0.002 |
| Darman | 3.94 | ± 0.011 | 4.35 | | ± 0.117 |  | 0.74 | ± 0.005 | 1.15 | ± 0.005 |
| DarrehLak | 3.80 | ± 0.043 | 3.83 | | ± 0.025 |  | 0.88 | ± 0.016 | 1.29 | ± 0.002 |
| DoSar | 3.97 | ± 0.005 | 4.07 | | ± 0.015 |  | 1.47 | ± 0.085 | 1.74 | ± 0.011 |
| Dowlatabad | 3.56 | ± 0.004 | 4.00 | | ± 0.015 |  | 0.92 | ± 0.025 | 1.39 | ± 0.073 |
| Duzduzan | 3.68 | ± 0.016 | 3.94 | | ± 0.024 |  | 1.01 | ± 0.045 | 1.32 | ± 0.035 |
| EisaKand | 3.80 | ± 0.025 | 3.98 | | ± 0.035 |  | 1.07 | ± 0.096 | 0.96 | ± 0.002 |
| Filabad | 3.36 | ± 0.025 | 3.91 | | ± 0.002 |  | 0.73 | ± 0.006 | 1.11 | ± 0.007 |
| Gaznaq | 3.59 | ± 0.043 | 3.92 | | ± 0.079 |  | 0.93 | ± 0.015 | 1.43 | ± 0.065 |
| Ghircanyon1 | 2.92 | ± 0.096 | 3.18 | | ± 0.046 |  | 0.67 | ± 0.021 | 0.97 | ± 0.014 |
| GilanTappeh | 3.43 | ± 0.018 | 3.89 | | ± 0.032 |  | 0.23 | ± 0.049 | 0.62 | ± 0.140 |
| Goorsephid | 3.78 | ± 0.037 | 4.10 | | ± 0.026 |  | 0.65 | ± 0.003 | 1.18 | ± 0.095 |
| Hamashahr | 3.54 | ± 0.040 | 3.97 | | ± 0.055 |  | 0.97 | ± 0.086 | 1.23 | ± 0.093 |
| Hamedan | 3.63 | ± 0.145 | 3.87 | | ± 0.143 |  | 1.46 | ± 0.018 | 1.63 | ± 0.003 |
| HasanQeshlaq | 3.19 | ± 0.005 | 3.92 | | ± 0.013 |  | 0.84 | ± 0.003 | 1.14 | ± 0.000 |
| Heyran | 3.44 | ± 0.046 | 3.82 | | ± 0.018 |  | 0.64 | ± 0.014 | 0.97 | ± 0.017 |
| Hezarkanian | 3.76 | ± 0.053 | 4.28 | | ± 0.041 |  | 1.24 | ± 0.137 | 1.58 | ± 0.011 |
| Isparaxan | 3.43 | ± 0.090 | 4.20 | | ± 0.005 |  | 0.98 | ± 0.007 | 1.04 | ± 0.024 |
| Kalat | 3.55 | ± 0.123 | 3.80 | | ± 0.144 |  | 0.78 | ± 0.030 | 1.09 | ± 0.009 |
| KalatehNaqi | 3.26 | ± 0.046 | 3.91 | | ± 0.044 |  | 0.79 | ± 0.038 | 1.18 | ± 0.086 |
| KaniGanji | 3.44 | ± 0.114 | 3.56 | | ± 0.030 |  | 0.97 | ± 0.074 | 1.30 | ± 0.003 |
| Kargan | 3.32 | ± 0.012 | 3.80 | | ± 0.028 |  | 0.97 | ± 0.116 | 1.19 | ± 0.081 |
| Karimabad | 3.17 | ± 0.005 | 3.20 | | ± 0.001 |  | 0.40 | ± 0.078 | 0.93 | ± 0.018 |
| Karkaraq | 3.11 | ± 0.012 | 3.53 | | ± 0.000 |  | 0.81 | ± 0.002 | 0.94 | ± 0.000 |
| Karvandan | 4.13 | ± 0.009 | 4.43 | | ± 0.009 |  | 1.46 | ± 0.016 | 1.73 | ± 0.042 |
| Kelardasht | 3.19 | ± 0.084 | 3.59 | | ± 0.021 |  | 0.69 | ± 0.032 | 1.16 | ± 0.134 |
| Khorramabad | 3.05 | ± 0.035 | 3.82 | | ± 0.108 |  | 0.74 | ± 0.060 | 1.07 | ± 0.002 |
| Khosrowabad | 3.16 | ± 0.005 | 3.64 | | ± 0.110 |  | 0.83 | ± 0.066 | 1.22 | ± 0.082 |
| KusehKahriz | 3.39 | ± 0.028 | 3.51 | | ± 0.008 |  | 0.85 | ± 0.039 | 0.93 | ± 0.001 |
| Laj | 3.65 | ± 0.118 | 3.83 | | ± 0.034 |  | 0.75 | ± 0.072 | 1.10 | ± 0.030 |
| Lamis | 3.38 | ± 0.004 | 3.99 | | ± 0.044 |  | 0.83 | ± 0.013 | 1.17 | ± 0.090 |
| LasemCheshmeh | 3.80 | ± 0.045 | 4.03 | | ± 0.019 |  | 1.32 | ± 0.046 | 1.29 | ± 0.031 |
| Liqvan | 3.92 | ± 0.013 | 4.51 | | ± 0.006 |  | 0.67 | ± 0.046 | 0.97 | ± 0.023 |
| Losku | 2.75 | ± 0.023 | 3.05 | | ± 0.077 |  | 0.46 | ± 0.132 | 0.71 | ± 0.003 |
| Mamlejeh | 3.26 | ± 0.056 | 3.59 | | ± 0.036 |  | 0.79 | ± 0.046 | 1.17 | ± 0.091 |
| Marian | 3.02 | ± 0.015 | 0.38 | | ± 0.013 |  | 0.58 | ± 0.041 | 0.91 | ± 0.004 |
| MazraeBeed | 2.79 | ± 0.077 | 3.01 | | ± 0.012 |  | 0.71 | ± 0.014 | 1.13 | ± 0.128 |
| MihamlehyeOlya | 3.93 | ± 0.022 | 4.47 | | ± 0.008 |  | 0.84 | ± 0.004 | 1.20 | ± 0.001 |
| MirAzizi | 3.60 | ± 0.056 | 3.75 | | ± 0.048 |  | 0.77 | ± 0.018 | 1.05 | ± 0.034 |
| Naharkhoran | 3.17 | ± 0.010 | 3.41 | | ± 0.037 |  | 1.17 | ± 0.189 | 1.15 | ± 0.104 |
| Nasirabad | 3.30 | ± 0.001 | 3.66 | | ± 0.038 |  | 0.65 | ± 0.011 | 0.93 | ± 0.000 |
| Noqan | 3.91 | ± 0.056 | 4.43 | | ± 0.006 |  | 0.65 | ± 0.008 | 1.19 | ± 0.001 |
| Nowgaran | 3.26 | ± 0.043 | 3.47 | | ± 0.045 |  | 0.72 | ± 0.031 | 1.03 | ± 0.016 |
| PahnehBar | 3.27 | ± 0.051 | 3.62 | | ± 0.027 |  | 1.05 | ± 0.010 | 0.82 | ± 0.023 |
| Palam | 3.32 | ± 0.104 | 3.56 | | ± 0.092 |  | 0.97 | ± 0.072 | 1.27 | ± 0.022 |
| Qorveh | 3.22 | ± 0.004 | 3.73 | | ± 0.036 |  | 0.92 | ± 0.063 | 1.27 | ± 0.022 |
| Qozivand | 3.40 | ± 0.077 | 3.53 | | ± 0.020 |  | 0.80 | ± 0.004 | 1.06 | ± 0.034 |
| QuriChay | 3.32 | ± 0.005 | 3.97 | | ± 0.006 |  | 1.01 | ± 0.023 | 0.70 | ± 0.021 |
| RezaqoliyeQeshlaq | 3.07 | ± 0.005 | 4.00 | | ± 0.019 |  | 0.93 | ± 0.039 | 0.93 | ± 0.002 |
| Roodafshan | 3.21 | ± 0.047 | 3.49 | | ± 0.013 |  | 0.77 | ± 0.016 | 1.03 | ± 0.035 |
| Sarab | 3.92 | ± 0.005 | 4.13 | | ± 0.091 |  | 0.94 | ± 0.017 | 0.93 | ± 0.018 |
| Sarbandan | 3.29 | ± 0.003 | 3.64 | | ± 0.051 |  | 0.60 | ± 0.017 | 0.96 | ± 0.024 |
| Seranza | 3.11 | ± 0.008 | 3.69 | | ± 0.125 |  | 0.66 | ± 0.021 | 1.20 | ± 0.001 |
| Shamasbi | 3.43 | ± 0.102 | 3.96 | | ± 0.064 |  | 0.78 | ± 0.038 | 1.05 | ± 0.038 |
| TangeSehRiz1 | 3.35 | ± 0.054 | 3.79 | | ± 0.024 |  | 1.03 | ± 0.007 | 1.53 | ± 0.216 |
| Sileh | 3.25 | ± 0.169 | 4.01 | | ± 0.030 |  | 0.72 | ± 0.037 | 1.06 | ± 0.024 |
| SinavaCheshme | 2.76 | ± 0.010 | 3.46 | | ± 0.033 |  | 0.41 | ± 0.125 | 1.09 | ± 0.149 |
| Subashi | 3.79 | ± 0.052 | 4.03 | | ± 0.048 |  | 0.68 | ± 0.020 | 0.96 | ± 0.028 |
| Sureshjan | 3.21 | ± 0.121 | 3.53 | | ± 0.071 |  | 0.71 | ± 0.007 | 1.23 | ± 0.000 |
| Talesh | 2.73 | ± 0.025 | 3.03 | | ± 0.009 |  | 0.42 | ± 0.079 | 1.22 | ± 0.007 |
| TangeSehRiz2 | 3.24 | ± 0.008 | 3.95 | | ± 0.069 |  | 1.06 | ± 0.174 | 0.90 | ± 0.005 |
| TangeTizab | 3.16 | ± 0.025 | 3.52 | | ± 0.046 |  | 0.67 | ± 0.040 | 1.21 | ± 0.002 |
| Tangrah | 3.51 | ± 0.001 | 3.95 | | ± 0.075 |  | 0.84 | ± 0.027 | 1.04 | ± 0.012 |
| Tazehabad | 2.94 | ± 0.001 | 3.02 | | ± 0.006 |  | 0.27 | ± 0.090 | 0.68 | ± 0.019 |
| Telochal | 3.34 | ± 0.154 | 3.51 | | ± 0.015 |  | 0.69 | ± 0.032 | 0.90 | ± 0.035 |
| Tokhmaqlu | 3.10 | ± 0.004 | 3.80 | | ± 0.074 |  | 0.99 | ± 0.043 | 1.13 | ± 0.111 |
| Torshab | 3.92 | ± 0.044 | 4.10 | | ± 0.005 |  | 0.89 | ± 0.041 | 0.92 | ± 0.001 |
| Vanehbin | 3.17 | ± 0.058 | 3.73 | | ± 0.035 |  | 0.79 | ± 0.011 | 1.17 | ± 0.064 |
| Vila Darre Waterfall | 3.68 | ± 0.055 | 3.95 | | ± 0.035 |  | 1.24 | ± 0.000 | 1.65 | ± 0.008 |
| Yasuj | 4.01 | ± 0.114 | 3.27 | | ± 0.015 |  | 0.74 | ± 0.064 | 1.01 | ± 0.007 |
| Ziarat | 3.25 | ± 0.020 | 3.53 | | ± 0.065 |  | 0.62 | ± 0.011 | 0.89 | ± 0.024 |
| G | Non-stress | | | | |  | Drought stress | | | |
|  | 2018 | | 2019 | | |  | 2018 | | 2019 | |
|  | Chl | ± SE | Chl | | ± SE |  | Chl | ± SE | Chl | ± SE |
| Abbasabad | 9.18 | ± 0.023 | 9.63 | ± 0.029 | |  | 5.81 | ± 0.006 | 6.35 | ± 0.117 |
| Abr1 | 9.70 | ± 0.090 | 10.80 | ± 0.126 | |  | 5.91 | ± 0.040 | 6.61 | ± 0.037 |
| Abr1Forest1 | 9.89 | ± 0.055 | 10.83 | ± 0.015 | |  | 7.04 | ± 0.027 | 7.39 | ± 0.003 |
| Abr2 | 10.51 | ± 0.447 | 12.00 | ± 0.038 | |  | 7.72 | ± 0.071 | 7.95 | ± 0.291 |
| Abr2Forest2 | 9.44 | ± 0.221 | 10.29 | ± 0.236 | |  | 6.89 | ± 0.378 | 7.25 | ± 0.037 |
| Abr3Forest3 | 9.80 | ± 0.203 | 10.69 | ± 0.221 | |  | 6.66 | ± 0.019 | 7.21 | ± 0.061 |
| Abrumand | 10.62 | ± 0.070 | 11.56 | ± 0.090 | |  | 7.90 | ± 0.190 | 8.46 | ± 0.335 |
| Ahangaran | 10.54 | ± 0.123 | 10.77 | ± 0.203 | |  | 6.79 | ± 0.051 | 7.25 | ± 0.083 |
| Alvaresi | 10.64 | ± 0.081 | 11.12 | ± 0.153 | |  | 7.01 | ± 0.251 | 7.53 | ± 0.002 |
| Asadli | 10.62 | ± 0.100 | 10.75 | ± 0.091 | |  | 7.53 | ± 0.062 | 7.52 | ± 0.095 |
| Ashab | 9.10 | ± 0.036 | 9.78 | ± 0.161 | |  | 6.62 | ± 0.557 | 7.43 | ± 0.089 |
| Azizabad | 10.63 | ± 0.114 | 10.89 | ± 0.068 | |  | 6.71 | ± 0.388 | 7.22 | ± 0.240 |
| Aznãvleh | 9.82 | ± 0.150 | 10.33 | ± 0.087 | |  | 6.21 | ± 0.051 | 7.67 | ± 0.285 |
| BadKhoreh | 10.19 | ± 0.051 | 10.40 | ± 0.127 | |  | 7.10 | ± 0.058 | 7.22 | ± 0.273 |
| BandarehAnzali | 9.51 | ± 0.040 | 9.90 | ± 0.059 | |  | 6.05 | ± 0.028 | 6.85 | ± 0.048 |
| Baneh | 9.89 | ± 0.078 | 10.51 | ± 0.152 | |  | 7.19 | ± 0.083 | 7.71 | ± 0.006 |
| Basmenj | 10.68 | ± 0.062 | 10.91 | ± 0.044 | |  | 7.52 | ± 0.131 | 7.51 | ± 0.006 |
| Beyraq | 9.66 | ± 0.307 | 10.54 | ± 0.335 | |  | 7.20 | ± 0.034 | 7.05 | ± 0.011 |
| Bisotun | 9.85 | ± 0.406 | 10.30 | ± 0.001 | |  | 7.10 | ± 0.071 | 6.99 | ± 0.007 |
| Borbor | 10.12 | ± 0.211 | 10.77 | ± 0.041 | |  | 7.09 | ± 0.033 | 7.53 | ± 0.089 |
| Borhan | 10.62 | ± 0.025 | 9.82 | ± 0.165 | |  | 6.36 | ± 0.010 | 7.96 | ± 0.035 |
| Chadegan | 9.90 | ± 0.066 | 10.80 | ± 0.072 | |  | 6.54 | ± 0.036 | 7.20 | ± 0.023 |
| Chaleki | 9.36 | ± 0.103 | 9.73 | ± 0.036 | |  | 6.02 | ± 0.033 | 6.82 | ± 0.100 |
| Chali | 9.71 | ± 0.026 | 10.62 | ± 0.000 | |  | 6.50 | ± 0.351 | 7.21 | ± 0.175 |
| Chavarchin | 9.82 | ± 0.251 | 10.71 | ± 0.273 | |  | 7.17 | ± 0.070 | 7.44 | ± 0.038 |
| Ciakhor | 10.64 | ± 0.094 | 11.36 | ± 0.015 | |  | 7.70 | ± 0.034 | 8.13 | ± 0.064 |
| Damavand | 10.74 | ± 0.020 | 10.80 | ± 0.034 | |  | 7.72 | ± 0.045 | 8.18 | ± 0.001 |
| Damghanat | 9.94 | ± 0.127 | 10.84 | ± 0.139 | |  | 6.65 | ± 0.312 | 7.25 | ± 0.009 |
| Darband | 9.64 | ± 0.010 | 10.26 | ± 0.117 | |  | 6.81 | ± 0.319 | 7.07 | ± 0.008 |
| DareSari | 10.51 | ± 0.039 | 10.76 | ± 0.040 | |  | 6.89 | ± 0.526 | 7.10 | ± 0.014 |
| Darman | 10.79 | ± 0.058 | 9.88 | ± 0.077 | |  | 6.66 | ± 0.029 | 6.84 | ± 0.108 |
| DarrehLak | 9.68 | ± 0.063 | 10.56 | ± 0.069 | |  | 7.51 | ± 0.051 | 7.49 | ± 0.108 |
| DoSar | 10.53 | ± 0.095 | 10.76 | ± 0.046 | |  | 7.13 | ± 0.001 | 8.12 | ± 0.050 |
| Dowlatabad | 10.14 | ± 0.012 | 10.48 | ± 0.126 | |  | 7.27 | ± 0.108 | 7.72 | ± 0.016 |
| Duzduzan | 9.89 | ± 0.012 | 10.79 | ± 0.013 | |  | 6.96 | ± 0.425 | 7.71 | ± 0.008 |
| EisaKand | 9.72 | ± 0.128 | 10.60 | ± 0.139 | |  | 6.81 | ± 0.341 | 7.90 | ± 0.083 |
| Filabad | 9.75 | ± 0.120 | 10.77 | ± 0.005 | |  | 6.66 | ± 0.024 | 7.22 | ± 0.005 |
| Gaznaq | 10.14 | ± 0.043 | 10.70 | ± 0.130 | |  | 6.85 | ± 0.516 | 7.68 | ± 0.055 |
| Ghircanyon1 | 9.46 | ± 0.094 | 9.86 | ± 0.091 | |  | 6.68 | ± 0.549 | 7.30 | ± 0.247 |
| GilanTappeh | 9.79 | ± 0.050 | 10.36 | ± 0.230 | |  | 5.75 | ± 0.031 | 6.62 | ± 0.184 |
| Goorsephid | 10.40 | ± 0.039 | 10.72 | ± 0.148 | |  | 6.70 | ± 0.497 | 7.33 | ± 0.033 |
| Hamashahr | 10.08 | ± 0.114 | 10.77 | ± 0.008 | |  | 7.57 | ± 0.000 | 7.55 | ± 0.172 |
| Hamedan | 10.06 | ± 0.144 | 10.70 | ± 0.018 | |  | 7.69 | ± 0.080 | 8.01 | ± 0.028 |
| HasanQeshlaq | 9.91 | ± 0.053 | 10.78 | ± 0.035 | |  | 7.55 | ± 0.025 | 7.84 | ± 0.026 |
| Heyran | 9.79 | ± 0.131 | 10.52 | ± 0.050 | |  | 6.70 | ± 0.534 | 7.34 | ± 0.228 |
| Hezarkanian | 10.69 | ± 0.011 | 9.97 | ± 0.002 | |  | 7.63 | ± 0.014 | 7.88 | ± 0.060 |
| Isparaxan | 9.70 | ± 0.043 | 10.71 | ± 0.007 | |  | 6.42 | ± 0.014 | 7.17 | ± 0.007 |
| Kalat | 9.87 | ± 0.089 | 10.46 | ± 0.395 | |  | 6.95 | ± 0.105 | 7.19 | ± 0.151 |
| KalatehNaqi | 9.89 | ± 0.110 | 10.78 | ± 0.120 | |  | 6.77 | ± 0.056 | 7.37 | ± 0.019 |
| KaniGanji | 9.77 | ± 0.358 | 10.31 | ± 0.044 | |  | 6.38 | ± 0.099 | 7.70 | ± 0.000 |
| Kargan | 9.52 | ± 0.017 | 10.48 | ± 0.077 | |  | 6.81 | ± 0.348 | 7.21 | ± 0.171 |
| Karimabad | 9.58 | ± 0.044 | 10.03 | ± 0.032 | |  | 5.97 | ± 0.030 | 6.88 | ± 0.029 |
| Karkaraq | 9.52 | ± 0.018 | 10.38 | ± 0.020 | |  | 6.75 | ± 0.194 | 7.43 | ± 0.010 |
| Karvandan | 10.36 | ± 0.189 | 11.17 | ± 0.103 | |  | 7.63 | ± 0.001 | 8.09 | ± 0.013 |
| Kelardasht | 9.48 | ± 0.031 | 10.34 | ± 0.034 | |  | 7.02 | ± 0.250 | 7.28 | ± 0.028 |
| Khorramabad | 9.93 | ± 0.073 | 10.55 | ± 0.102 | |  | 7.34 | ± 0.016 | 7.24 | ± 0.073 |
| Khosrowabad | 9.57 | ± 0.028 | 10.64 | ± 0.236 | |  | 7.08 | ± 0.044 | 7.44 | ± 0.024 |
| KusehKahriz | 10.09 | ± 0.021 | 9.54 | ± 0.103 | |  | 7.01 | ± 0.071 | 7.29 | ± 0.285 |
| Laj | 9.92 | ± 0.156 | 10.55 | ± 0.093 | |  | 7.01 | ± 0.341 | 7.29 | ± 0.003 |
| Lamis | 9.62 | ± 0.010 | 10.80 | ± 0.071 | |  | 6.84 | ± 0.130 | 7.23 | ± 0.190 |
| LasemCheshmeh | 10.66 | ± 0.158 | 9.62 | ± 0.006 | |  | 7.28 | ± 0.156 | 7.40 | ± 0.371 |
| Liqvan | 10.99 | ± 0.040 | 11.32 | ± 0.053 | |  | 6.86 | ± 0.326 | 7.08 | ± 0.030 |
| Losku | 9.41 | ± 0.250 | 9.67 | ± 0.051 | |  | 6.09 | ± 0.050 | 6.53 | ± 0.006 |
| Mamlejeh | 9.54 | ± 0.020 | 10.41 | ± 0.022 | |  | 6.87 | ± 0.120 | 7.27 | ± 0.103 |
| Marian | 10.32 | ± 0.040 | 9.68 | ± 0.061 | |  | 6.09 | ± 0.141 | 6.98 | ± 0.089 |
| MazraeBeed | 9.15 | ± 0.001 | 9.63 | ± 0.006 | |  | 6.66 | ± 0.057 | 7.38 | ± 0.150 |
| MihamlehyeOlya | 10.61 | ± 0.075 | 11.11 | ± 0.261 | |  | 6.91 | ± 0.162 | 7.09 | ± 0.022 |
| MirAzizi | 9.84 | ± 0.259 | 10.32 | ± 0.133 | |  | 6.41 | ± 0.357 | 7.34 | ± 0.005 |
| Naharkhoran | 9.48 | ± 0.125 | 10.33 | ± 0.136 | |  | 7.62 | ± 0.009 | 7.46 | ± 0.006 |
| Nasirabad | 9.42 | ± 0.001 | 10.85 | ± 0.078 | |  | 6.21 | ± 0.007 | 7.02 | ± 0.022 |
| Noqan | 10.61 | ± 0.004 | 11.22 | ± 0.051 | |  | 6.25 | ± 0.013 | 7.33 | ± 0.200 |
| Nowgaran | 10.22 | ± 0.050 | 9.76 | ± 0.036 | |  | 6.99 | ± 0.285 | 7.15 | ± 0.011 |
| PahnehBar | 9.48 | ± 0.026 | 10.34 | ± 0.028 | |  | 6.68 | ± 0.370 | 6.61 | ± 0.065 |
| Palam | 9.49 | ± 0.265 | 10.29 | ± 0.229 | |  | 7.30 | ± 0.155 | 6.98 | ± 0.011 |
| Qorveh | 9.41 | ± 0.091 | 10.27 | ± 0.099 | |  | 7.27 | ± 0.101 | 7.64 | ± 0.039 |
| Qozivand | 9.51 | ± 0.092 | 10.37 | ± 0.100 | |  | 6.97 | ± 0.015 | 7.28 | ± 0.029 |
| QuriChay | 9.86 | ± 0.090 | 10.40 | ± 0.252 | |  | 6.81 | ± 0.466 | 6.48 | ± 0.014 |
| RezaqoliyeQeshlaq | 9.75 | ± 0.151 | 10.63 | ± 0.164 | |  | 6.77 | ± 0.298 | 7.01 | ± 0.023 |
| Roodafshan | 10.11 | ± 0.042 | 9.59 | ± 0.019 | |  | 6.44 | ± 0.270 | 7.04 | ± 0.126 |
| Sarab | 10.41 | ± 0.057 | 12.02 | ± 0.037 | |  | 6.38 | ± 0.053 | 7.23 | ± 0.291 |
| Sarbandan | 9.39 | ± 0.076 | 10.24 | ± 0.083 | |  | 6.46 | ± 0.244 | 7.30 | ± 0.227 |
| Seranza | 9.54 | ± 0.111 | 9.77 | ± 0.100 | |  | 7.09 | ± 0.179 | 7.91 | ± 0.004 |
| Shamasbi | 9.96 | ± 0.114 | 10.75 | ± 0.015 | |  | 6.61 | ± 0.437 | 7.09 | ± 0.116 |
| TangeSehRiz1 | 9.78 | ± 0.040 | 10.15 | ± 0.114 | |  | 6.85 | ± 0.508 | 7.84 | ± 0.004 |
| Sileh | 9.86 | ± 0.109 | 10.72 | ± 0.066 | |  | 6.72 | ± 0.576 | 7.23 | ± 0.051 |
| SinavaCheshme | 9.31 | ± 0.055 | 10.08 | ± 0.083 | |  | 6.37 | ± 0.329 | 7.12 | ± 0.008 |
| Subashi | 10.49 | ± 0.025 | 10.93 | ± 0.028 | |  | 6.77 | ± 0.405 | 7.07 | ± 0.027 |
| Sureshjan | 9.57 | ± 0.077 | 10.52 | ± 0.005 | |  | 6.66 | ± 0.091 | 7.33 | ± 0.236 |
| Talesh | 9.27 | ± 0.101 | 9.78 | ± 0.045 | |  | 5.90 | ± 0.114 | 6.73 | ± 0.011 |
| TangeSehRiz2 | 9.73 | ± 0.062 | 10.62 | ± 0.067 | |  | 7.22 | ± 0.142 | 6.94 | ± 0.200 |
| TangeTizab | 9.49 | ± 0.025 | 10.35 | ± 0.027 | |  | 6.87 | ± 0.293 | 7.56 | ± 0.009 |
| Tangrah | 9.96 | ± 0.044 | 10.74 | ± 0.075 | |  | 6.93 | ± 0.237 | 7.16 | ± 0.002 |
| Tazehabad | 9.43 | ± 0.164 | 9.89 | ± 0.052 | |  | 5.90 | ± 0.035 | 6.40 | ± 0.052 |
| Telochal | 9.78 | ± 0.178 | 10.44 | ± 0.037 | |  | 6.43 | ± 0.245 | 7.23 | ± 0.275 |
| Tokhmaqlu | 9.43 | ± 0.059 | 9.72 | ± 0.075 | |  | 6.47 | ± 0.059 | 7.48 | ± 0.020 |
| Torshab | 10.57 | ± 0.028 | 10.90 | ± 0.065 | |  | 7.45 | ± 0.014 | 7.73 | ± 0.093 |
| Vanehbin | 9.42 | ± 0.087 | 10.28 | ± 0.095 | |  | 6.79 | ± 0.252 | 7.19 | ± 0.018 |
| Vila Darre Waterfall | 9.98 | ± 0.087 | 10.88 | ± 0.095 | |  | 7.38 | ± 0.025 | 7.75 | ± 0.004 |
| Yasuj | 10.32 | ± 0.053 | 10.10 | ± 0.064 | |  | 7.05 | ± 0.224 | 7.16 | ± 0.024 |
| Ziarat | 9.41 | ± 0.109 | 10.27 | ± 0.118 | |  | 6.51 | ± 0.360 | 7.03 | ± 0.195 |
| G | Non-stress | | | | |  | Drought stress | | | |
|  | 2018 | | 2019 | | |  | 2018 | | 2019 | |
|  | Fv/Fm | ± SE | Fv/Fm | | ± SE |  | Fv/Fm | ± SE | Fv/Fm | ± SE |
| Abbasabad | 0.68 | ± 0.0025 | 0.68 | | ± 0.0220 |  | 0.38 | ± 0.0045 | 0.49 | ± 0.0060 |
| Abr1 | 0.67 | ± 0.0075 | 0.72 | | ± 0.0080 |  | 0.39 | ± 0.0040 | 0.52 | ± 0.0040 |
| Abr1Forest1 | 0.69 | ± 0.0040 | 0.75 | | ± 0.0045 |  | 0.57 | ± 0.0090 | 0.59 | ± 0.0125 |
| Abr2 | 0.80 | ± 0.0055 | 0.82 | | ± 0.0040 |  | 0.62 | ± 0.0240 | 0.65 | ± 0.0260 |
| Abr2Forest2 | 0.66 | ± 0.0225 | 0.71 | | ± 0.0240 |  | 0.55 | ± 0.0030 | 0.59 | ± 0.0155 |
| Abr3Forest3 | 0.67 | ± 0.0080 | 0.72 | | ± 0.0085 |  | 0.51 | ± 0.0085 | 0.54 | ± 0.0130 |
| Abrumand | 0.79 | ± 0.0050 | 0.84 | | ± 0.0005 |  | 0.68 | ± 0.0160 | 0.73 | ± 0.0000 |
| Ahangaran | 0.74 | ± 0.0085 | 0.78 | | ± 0.0070 |  | 0.53 | ± 0.0170 | 0.56 | ± 0.0070 |
| Alvaresi | 0.77 | ± 0.0155 | 0.82 | | ± 0.0070 |  | 0.51 | ± 0.0460 | 0.56 | ± 0.0015 |
| Asadli | 0.75 | ± 0.0070 | 0.78 | | ± 0.0065 |  | 0.61 | ± 0.0010 | 0.64 | ± 0.0013 |
| Ashab | 0.67 | ± 0.0060 | 0.75 | | ± 0.0100 |  | 0.50 | ± 0.0595 | 0.59 | ± 0.0169 |
| Azizabad | 0.75 | ± 0.0080 | 0.78 | | ± 0.0050 |  | 0.53 | ± 0.0335 | 0.60 | ± 0.0105 |
| Aznãvleh | 0.70 | ± 0.0010 | 0.75 | | ± 0.0005 |  | 0.50 | ± 0.0515 | 0.63 | ± 0.0080 |
| BadKhoreh | 0.68 | ± 0.0010 | 0.73 | | ± 0.0015 |  | 0.55 | ± 0.0080 | 0.55 | ± 0.0117 |
| BandarehAnzali | 0.70 | ± 0.0005 | 0.73 | | ± 0.0165 |  | 0.43 | ± 0.0155 | 0.53 | ± 0.0015 |
| Baneh | 0.69 | ± 0.0055 | 0.75 | | ± 0.0055 |  | 0.59 | ± 0.0020 | 0.67 | ± 0.0005 |
| Basmenj | 0.77 | ± 0.0005 | 0.80 | | ± 0.0210 |  | 0.58 | ± 0.0210 | 0.67 | ± 0.0010 |
| Beyraq | 0.77 | ± 0.0100 | 0.78 | | ± 0.0045 |  | 0.49 | ± 0.0330 | 0.57 | ± 0.0234 |
| Bisotun | 0.72 | ± 0.0035 | 0.77 | | ± 0.0090 |  | 0.50 | ± 0.0140 | 0.61 | ± 0.0340 |
| Borbor | 0.73 | ± 0.0080 | 0.77 | | ± 0.0070 |  | 0.52 | ± 0.0230 | 0.59 | ± 0.0380 |
| Borhan | 0.76 | ± 0.0045 | 0.77 | | ± 0.0070 |  | 0.58 | ± 0.0040 | 0.67 | ± 0.0095 |
| Chadegan | 0.75 | ± 0.0010 | 0.77 | | ± 0.0175 |  | 0.54 | ± 0.0155 | 0.57 | ± 0.0005 |
| Chaleki | 0.69 | ± 0.0105 | 0.79 | | ± 0.0100 |  | 0.42 | ± 0.0110 | 0.53 | ± 0.0122 |
| Chali | 0.67 | ± 0.0100 | 0.72 | | ± 0.0110 |  | 0.51 | ± 0.0000 | 0.60 | ± 0.0095 |
| Chavarchin | 0.74 | ± 0.0025 | 0.78 | | ± 0.0205 |  | 0.56 | ± 0.0205 | 0.60 | ± 0.0058 |
| Ciakhor | 0.80 | ± 0.0025 | 0.85 | | ± 0.0050 |  | 0.62 | ± 0.0190 | 0.71 | ± 0.0090 |
| Damavand | 0.78 | ± 0.0200 | 0.82 | | ± 0.0165 |  | 0.64 | ± 0.0045 | 0.72 | ± 0.0090 |
| Damghanat | 0.75 | ± 0.0205 | 0.76 | | ± 0.0165 |  | 0.50 | ± 0.0120 | 0.59 | ± 0.0040 |
| Darband | 0.68 | ± 0.0005 | 0.73 | | ± 0.0005 |  | 0.55 | ± 0.0155 | 0.57 | ± 0.0125 |
| DareSari | 0.77 | ± 0.0180 | 0.79 | | ± 0.0035 |  | 0.53 | ± 0.0180 | 0.62 | ± 0.0070 |
| Darman | 0.75 | ± 0.0050 | 0.79 | | ± 0.0060 |  | 0.53 | ± 0.0100 | 0.52 | ± 0.0020 |
| DarrehLak | 0.76 | ± 0.0085 | 0.78 | | ± 0.0170 |  | 0.66 | ± 0.0240 | 0.63 | ± 0.0009 |
| DoSar | 0.77 | ± 0.0030 | 0.78 | | ± 0.0015 |  | 0.62 | ± 0.0235 | 0.71 | ± 0.0135 |
| Dowlatabad | 0.71 | ± 0.0005 | 0.77 | | ± 0.0010 |  | 0.53 | ± 0.0380 | 0.64 | ± 0.0285 |
| Duzduzan | 0.74 | ± 0.0030 | 0.77 | | ± 0.0210 |  | 0.58 | ± 0.0015 | 0.67 | ± 0.0390 |
| EisaKand | 0.76 | ± 0.0050 | 0.76 | | ± 0.0145 |  | 0.56 | ± 0.0355 | 0.60 | ± 0.0385 |
| Filabad | 0.67 | ± 0.0050 | 0.72 | | ± 0.0055 |  | 0.54 | ± 0.0170 | 0.59 | ± 0.0141 |
| Gaznaq | 0.72 | ± 0.0090 | 0.77 | | ± 0.0050 |  | 0.58 | ± 0.0015 | 0.63 | ± 0.0043 |
| Ghircanyon1 | 0.72 | ± 0.0030 | 0.73 | | ± 0.0160 |  | 0.48 | ± 0.0260 | 0.56 | ± 0.0077 |
| GilanTappeh | 0.69 | ± 0.0220 | 0.74 | | ± 0.0035 |  | 0.39 | ± 0.0320 | 0.49 | ± 0.0275 |
| Goorsephid | 0.72 | ± 0.0305 | 0.78 | | ± 0.0310 |  | 0.47 | ± 0.0340 | 0.57 | ± 0.0195 |
| Hamashahr | 0.71 | ± 0.0080 | 0.76 | | ± 0.0085 |  | 0.53 | ± 0.0430 | 0.64 | ± 0.0062 |
| Hamedan | 0.73 | ± 0.0290 | 0.76 | | ± 0.0140 |  | 0.64 | ± 0.0095 | 0.71 | ± 0.0100 |
| HasanQeshlaq | 0.64 | ± 0.0010 | 0.69 | | ± 0.0010 |  | 0.64 | ± 0.0250 | 0.69 | ± 0.0130 |
| Heyran | 0.69 | ± 0.0095 | 0.74 | | ± 0.0100 |  | 0.48 | ± 0.0230 | 0.59 | ± 0.0280 |
| Hezarkanian | 0.77 | ± 0.0115 | 0.79 | | ± 0.0070 |  | 0.61 | ± 0.0265 | 0.68 | ± 0.0040 |
| Isparaxan | 0.70 | ± 0.0090 | 0.79 | | ± 0.0020 |  | 0.58 | ± 0.0050 | 0.56 | ± 0.0015 |
| Kalat | 0.71 | ± 0.0245 | 0.77 | | ± 0.0265 |  | 0.55 | ± 0.0420 | 0.59 | ± 0.0005 |
| KalatehNaqi | 0.65 | ± 0.0095 | 0.70 | | ± 0.0095 |  | 0.56 | ± 0.0300 | 0.59 | ± 0.0035 |
| KaniGanji | 0.69 | ± 0.0230 | 0.74 | | ± 0.0245 |  | 0.54 | ± 0.0450 | 0.64 | ± 0.0015 |
| Kargan | 0.66 | ± 0.0025 | 0.72 | | ± 0.0025 |  | 0.58 | ± 0.0030 | 0.62 | ± 0.0112 |
| Karimabad | 0.73 | ± 0.0010 | 0.73 | | ± 0.0050 |  | 0.42 | ± 0.0035 | 0.52 | ± 0.0193 |
| Karkaraq | 0.62 | ± 0.0020 | 0.67 | | ± 0.0025 |  | 0.52 | ± 0.0025 | 0.63 | ± 0.0240 |
| Karvandan | 0.76 | ± 0.0005 | 0.83 | | ± 0.0100 |  | 0.64 | ± 0.0180 | 0.73 | ± 0.0115 |
| Kelardasht | 0.64 | ± 0.0170 | 0.69 | | ± 0.0180 |  | 0.52 | ± 0.0175 | 0.57 | ± 0.0145 |
| Khorramabad | 0.70 | ± 0.0050 | 0.75 | | ± 0.0055 |  | 0.50 | ± 0.0585 | 0.59 | ± 0.0100 |
| Khosrowabad | 0.63 | ± 0.0010 | 0.68 | | ± 0.0010 |  | 0.52 | ± 0.0220 | 0.63 | ± 0.0215 |
| KusehKahriz | 0.75 | ± 0.0100 | 0.73 | | ± 0.0035 |  | 0.55 | ± 0.0150 | 0.55 | ± 0.0005 |
| Laj | 0.73 | ± 0.0235 | 0.77 | | ± 0.0075 |  | 0.48 | ± 0.0245 | 0.55 | ± 0.0000 |
| Lamis | 0.68 | ± 0.0010 | 0.73 | | ± 0.0005 |  | 0.53 | ± 0.0265 | 0.55 | ± 0.0010 |
| LasemCheshmeh | 0.78 | ± 0.0280 | 0.78 | | ± 0.0025 |  | 0.55 | ± 0.0135 | 0.64 | ± 0.0001 |
| Liqvan | 0.76 | ± 0.0060 | 0.82 | | ± 0.0280 |  | 0.54 | ± 0.0085 | 0.56 | ± 0.0165 |
| Losku | 0.64 | ± 0.0085 | 0.70 | | ± 0.0125 |  | 0.42 | ± 0.0040 | 0.49 | ± 0.0115 |
| Mamlejeh | 0.65 | ± 0.0115 | 0.70 | | ± 0.0125 |  | 0.50 | ± 0.0170 | 0.54 | ± 0.0000 |
| Marian | 0.71 | ± 0.0020 | 0.75 | | ± 0.0030 |  | 0.45 | ± 0.0085 | 0.54 | ± 0.0025 |
| MazraeBeed | 0.67 | ± 0.0050 | 0.69 | | ± 0.0065 |  | 0.48 | ± 0.0000 | 0.55 | ± 0.0055 |
| MihamlehyeOlya | 0.76 | ± 0.0110 | 0.83 | | ± 0.0105 |  | 0.48 | ± 0.0005 | 0.58 | ± 0.0423 |
| MirAzizi | 0.72 | ± 0.0110 | 0.78 | | ± 0.0120 |  | 0.48 | ± 0.0020 | 0.54 | ± 0.0010 |
| Naharkhoran | 0.63 | ± 0.0020 | 0.68 | | ± 0.0020 |  | 0.61 | ± 0.0425 | 0.58 | ± 0.0235 |
| Nasirabad | 0.66 | ± 0.0005 | 0.74 | | ± 0.0325 |  | 0.44 | ± 0.0035 | 0.57 | ± 0.0020 |
| Noqan | 0.77 | ± 0.0055 | 0.83 | | ± 0.0135 |  | 0.45 | ± 0.0040 | 0.54 | ± 0.0002 |
| Nowgaran | 0.74 | ± 0.0045 | 0.77 | | ± 0.0100 |  | 0.52 | ± 0.0175 | 0.56 | ± 0.0208 |
| PahnehBar | 0.65 | ± 0.0105 | 0.70 | | ± 0.0110 |  | 0.50 | ± 0.0285 | 0.52 | ± 0.0125 |
| Palam | 0.66 | ± 0.0205 | 0.71 | | ± 0.0225 |  | 0.58 | ± 0.0080 | 0.57 | ± 0.0144 |
| Qorveh | 0.64 | ± 0.0005 | 0.69 | | ± 0.0010 |  | 0.53 | ± 0.0535 | 0.63 | ± 0.0120 |
| Qozivand | 0.68 | ± 0.0150 | 0.73 | | ± 0.0165 |  | 0.51 | ± 0.0405 | 0.61 | ± 0.0310 |
| QuriChay | 0.66 | ± 0.0010 | 0.72 | | ± 0.0010 |  | 0.52 | ± 0.0475 | 0.51 | ± 0.0005 |
| RezaqoliyeQeshlaq | 0.61 | ± 0.0005 | 0.66 | | ± 0.0005 |  | 0.52 | ± 0.0550 | 0.61 | ± 0.0465 |
| Roodafshan | 0.75 | ± 0.0100 | 0.81 | | ± 0.0110 |  | 0.49 | ± 0.0265 | 0.56 | ± 0.0096 |
| Sarab | 0.76 | ± 0.0040 | 0.80 | | ± 0.0045 |  | 0.48 | ± 0.0155 | 0.57 | ± 0.0095 |
| Sarbandan | 0.66 | ± 0.0005 | 0.71 | | ± 0.0005 |  | 0.51 | ± 0.0425 | 0.56 | ± 0.0246 |
| Seranza | 0.72 | ± 0.0105 | 0.74 | | ± 0.0050 |  | 0.44 | ± 0.0140 | 0.53 | ± 0.0045 |
| Shamasbi | 0.69 | ± 0.0205 | 0.74 | | ± 0.0220 |  | 0.54 | ± 0.0410 | 0.59 | ± 0.0095 |
| TangeSehRiz1 | 0.76 | ± 0.0040 | 0.78 | | ± 0.0180 |  | 0.58 | ± 0.0070 | 0.68 | ± 0.0155 |
| Sileh | 0.65 | ± 0.0335 | 0.70 | | ± 0.0365 |  | 0.51 | ± 0.0170 | 0.56 | ± 0.0085 |
| SinavaCheshme | 0.68 | ± 0.0035 | 0.71 | | ± 0.0295 |  | 0.48 | ± 0.0465 | 0.55 | ± 0.0014 |
| Subashi | 0.75 | ± 0.0125 | 0.78 | | ± 0.0170 |  | 0.49 | ± 0.0230 | 0.59 | ± 0.0285 |
| Sureshjan | 0.64 | ± 0.0240 | 0.69 | | ± 0.0260 |  | 0.46 | ± 0.0010 | 0.59 | ± 0.0240 |
| Talesh | 0.66 | ± 0.0400 | 0.69 | | ± 0.0015 |  | 0.41 | ± 0.0155 | 0.49 | ± 0.0000 |
| TangeSehRiz2 | 0.65 | ± 0.0015 | 0.70 | | ± 0.0015 |  | 0.57 | ± 0.0005 | 0.52 | ± 0.0035 |
| TangeTizab | 0.63 | ± 0.0050 | 0.68 | | ± 0.0055 |  | 0.43 | ± 0.0295 | 0.59 | ± 0.0095 |
| Tangrah | 0.70 | ± 0.0005 | 0.76 | | ± 0.0000 |  | 0.54 | ± 0.0245 | 0.58 | ± 0.0099 |
| Tazehabad | 0.69 | ± 0.0020 | 0.70 | | ± 0.0035 |  | 0.40 | ± 0.0070 | 0.50 | ± 0.0250 |
| Telochal | 0.67 | ± 0.0305 | 0.72 | | ± 0.0330 |  | 0.42 | ± 0.0260 | 0.53 | ± 0.0069 |
| Tokhmaqlu | 0.73 | ± 0.0010 | 0.75 | | ± 0.0045 |  | 0.53 | ± 0.0380 | 0.63 | ± 0.0185 |
| Torshab | 0.72 | ± 0.0165 | 0.78 | | ± 0.0035 |  | 0.54 | ± 0.0385 | 0.67 | ± 0.0346 |
| Vanehbin | 0.63 | ± 0.0115 | 0.68 | | ± 0.0125 |  | 0.49 | ± 0.0255 | 0.57 | ± 0.0075 |
| Vila Darre Waterfall | 0.74 | ± 0.0110 | 0.77 | | ± 0.0065 |  | 0.60 | ± 0.0005 | 0.67 | ± 0.0170 |
| Yasuj | 0.78 | ± 0.0005 | 0.75 | | ± 0.0085 |  | 0.53 | ± 0.0235 | 0.58 | ± 0.0095 |
| Ziarat | 0.65 | ± 0.0040 | 0.70 | | ± 0.0040 |  | 0.44 | ± 0.0055 | 0.56 | ± 0.0360 |
| FY, fresh forage yield; DY, dry forage weight; A, net photosynthetic rate; gs, stomatal conductance; Tr, transpiration rate; Chl, chlorophyll content; Fv/Fm, photochemical efficiency; SE, standard error of the mean. | | | | | | | | | | |
